# An 11-qubit atom processor in silicon

**DOI:** 10.1038/s41586-025-09827-w

**Published:** 2025-12-17

**Authors:** Hermann Edlbauer, Junliang Wang, A. M. Saffat-Ee Huq, Ian Thorvaldson, Michael T. Jones, Saiful Haque Misha, William J. Pappas, Christian M. Moehle, Yu-Ling Hsueh, Henric Bornemann, Samuel K. Gorman, Yousun Chung, Joris G. Keizer, Ludwik Kranz, Michelle Y. Simmons

**Affiliations:** Silicon Quantum Computing Pty Ltd, UNSW Sydney, Sydney, New South Wales Australia

**Keywords:** Quantum information, Qubits

## Abstract

Phosphorus atoms in silicon represent a promising platform for quantum computing, as their nuclear spins exhibit coherence times over seconds^1,2^ with high-fidelity readout and single-qubit control^3^. By placing several phosphorus atoms within a radius of a few nanometres, they couple by means of the hyperfine interaction to a single, shared electron. Such a nuclear spin register enables high-fidelity multi-qubit control^4^ and the execution of small-scale quantum algorithms^5^. An important requirement for scaling up is the ability to extend high-fidelity entanglement non-locally across several spin registers. Here we address this challenge with an 11-qubit atom processor composed of two multi-nuclear spin registers that are linked by means of electron exchange interaction. Through the advancement of calibration and control protocols, we achieve single-qubit and multi-qubit gates with all fidelities ranging from 99.10% to 99.99%. By entangling all combinations of local and non-local nuclear-spin pairs, we map out the performance of the processor and achieve state-of-the-art Bell-state fidelities of up to 99.5%. We then generate Greenberger–Horne–Zeilinger (GHZ) states with an increasing number of qubits and show entanglement of up to eight nuclear spins. By establishing high-fidelity operation across interconnected nuclear spin registers, we realize a key milestone towards fault-tolerant quantum computation with atom processors.

## Abstract (web summary)

An 11-qubit atom processor comprising two precision-placed nuclear spin registers of phosphorus in silicon is shown to achieve state-of-the-art Bell-state fidelities of up to 99.5%.

## Main

The predominant material in modern classical computers, silicon, is also a strong contender for the practical implementation of quantum processors^3,6–8^. To unlock the promised computational benefits of quantum computing, the qubit count needs to scale while maintaining high operation fidelity and connectivity. In terms of qubit numbers, the lead is at present held by superconducting^9,10^, ion-trap^11^ and neutral-atom^12^ processors, which approach hundreds of interconnected qubits. Further scale-up faces platform-specific challenges related to manufacturing, control-systems miniaturization and materials engineering. In this context, silicon quantum processors are emerging as a promising platform owing to their small footprint and materials compatibility with industrial manufacturing^8,13,14^.

In semiconductor devices, the number of individual qubits is increasing, with gate-defined arrays hosting up to 16 quantum dots^14,15^. So far, however, no more than four interconnected spin qubits were used in the execution of quantum circuits owing to challenges associated with multi-qubit control^16–19^. In this context, quantum computing with precision-placed phosphorus atoms in silicon, which we refer to as the 14|15 platform (according to the respective positions in the periodic table), is attracting growing interest driven by industry-leading physical-level metrics^3^ with exceptional, second-long coherence times^2,20^. The 14|15 platform uses precision manufacturing^21^ to place individual phosphorus atoms in close proximity (≲3 nm) to each other, in which a single loaded electron exhibits a hyperfine interaction with several nuclei. Such spin registers provide a unique set of advantages: the shared electron naturally acts as an ancilla qubit enabling quantum non-demolition (QND) readout of the nuclear spins and native multi-qubit (Toffoli) gates^4,5^. Combined with recent advances in silicon purification with sub-200 ppm of ^29^Si (ref. ^22^), these features enabled nuclear–nuclear CZ operations with fidelities exceeding 99% and the execution of three-qubit algorithms on a single multi-spin register^5^.

To enable the scaling of the 14|15 platform, it is essential to develop fast interconnects between quantum processing nodes without compromising performance^23^. The coupling of spin qubits is achievable by various mechanisms, such as dipolar interaction^24^ or spin–photon conversion in superconducting cavities^25^. The fastest coupling mechanism is provided by exchange interaction, as demonstrated with a 0.8-ns $\sqrt{{\rm{SWAP}}}$ gate between atomic qubits in natural silicon^26^. Exchange gates on electron spins have also been implemented with gate-defined quantum dots in isotopically pure silicon with fidelities greater than 99% (refs. ^27–30^). Successful implementation of exchange gates in atom qubits have already been achieved in purified silicon-28 (ref. ^31^), yet the limited two-qubit gate fidelity challenges the applicability of quantum-error-correction protocols^32,33^.

Here we report a precision-placed 11-qubit atom processor in isotopically purified silicon-28 that runs on a fast and efficient exchange-based link. Compared with the previous atom-based implementations with nuclear spin qubits^4,5,22^, we triple the number of coupled data qubits while maintaining the performance of single-qubit and two-qubit gates well above 99% fidelity. This achievement is enabled by systematic investigations of qubit stability, contextual errors and crosstalk, which informed the development of scalable calibration and control protocols. After outlining the basic set-up of the 11-qubit atom processor, we report the key metrics of single-qubit and two-qubit gates, assess pairwise entanglement for all combinations of nuclear spins and benchmark all-to-all connectivity through multi-qubit entanglement.

**Fig. 1:**
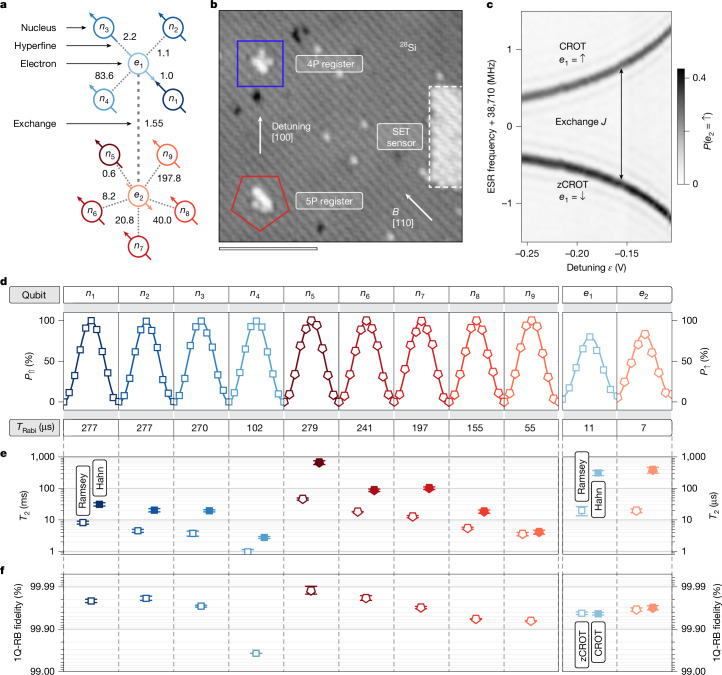
Single-qubit characteristics of the 11-qubit atom processor. **a**, Connectivity of nuclear spins (*n*_1_–*n*_9_) and electron spins (*e*_1_ and *e*_2_) through hyperfine and exchange coupling with energies in MHz. **b**, Scanning tunnelling micrograph of the processor core after hydrogen lithography showing the 4P register hosting *n*_1_–*n*_4_ and *e*_1_ (square) and the 5P register hosting *n*_5_–*n*_9_ and *e*_2_ (pentagon). The distance 13(1) nm (centre to centre) between the nuclear spin registers is atomically engineered to enable exchange coupling^26,54^. Scale bar, 10 nm. **c**, Exchange-coupled ESR spectrum of *e*_2_ as a function of voltage detuning *ε* with indications on the resonance frequencies corresponding to CROT and zCROT. **d**, Rabi oscillations along one period *T*_Rabi_ for all spins of the processor. We measure the spin-up probability of the nucleus *P*_⇑_ (electron *P*_*↑*_) as a function of the coherent NMR (ESR) drive duration. **e**, Phase coherence times measured for each spin through Ramsey (open symbols) and Hahn-echo (filled symbols) measurements. **f**, 1Q-RB results for each qubit showing average physical gate fidelities. SET, single-electron transistor.

The connectivity of the nuclei and electrons both within each register and across registers is central to the operation of the 11-qubit atom processor (Fig. 1a). Each spin register contains nuclei (*n*_1_–*n*_4_ and *n*_5_–*n*_9_) that are hyperfine-coupled to a common electron (*e*_1_ and *e*_2_). Notably, these electrons are also exchange-coupled to each other, enabling non-local connectivity across the registers (Fig. 1b). The strength of electron exchange coupling *J* is tunable by the voltage detuning *ε* across in-plane control gates (Fig. 1c and Supplementary Information Section I). The Hamiltonian of the system is described in Supplementary Information Section II. Here we operate in a weak exchange-coupled regime with *J* ≈ 1.55 MHz (Fig. 1c). In this regime, the controlled rotations (CROT) on the electron are less susceptible to charge noise and not conditional on the nuclear spins in the other register^26,34–36^. We note that the CROT operation on the electron spin has the advantage of implementing a native multi-qubit Toffoli gate that is conditional on the nuclear spins.

The initial calibration of the 11-qubit atom processor requires the characterization of 2^4^ + 2^5^ = 48 electron spin resonances (ESRs), which is doubled to 96 in the presence of electron exchange interaction. Analysing the stability of the ESR peaks (Supplementary Information Section III), we find that the frequencies within each register shift collectively. Accordingly, we can implement an efficient recalibration protocol that scales linearly with the number of coupled spin registers. By characterizing the ESR frequency for a single reference configuration of the nuclear spins, we infer the exact positions of all other ESR transitions of the register from the frequency offsets of the initial calibration. As a result, recalibrating all 96 ESR frequencies requires only two measurements, that is, one per register.

The state of the individual nuclear spins is controlled using nuclear magnetic resonance (NMR), similar to molecules in solution^37^ and nitrogen-vacancy centres in diamond^38,39^. The readout of an individual nuclear spin is performed through QND readout using the ancillary electron (Supplementary Information Section IV). For nuclear spin initialization, we combine this ESR-based approach with conditional NMR π pulses (Supplementary Information Section V). To maximize the fidelity of the initialized state, we perform QND readout of the nuclear spin configuration of each register before each experiment and apply post-selection on the desired nuclear spin configuration (Supplementary Information Section VI). For all experiments, unless stated otherwise, spectator qubits—that is, spins not actively involved in a given gate or quantum circuit—are initialized in the ⇓ state, and spin manipulations are performed conditional on these initialized states. The large contrast observed in Rabi oscillations (Fig. 1d) across all data qubits shows the performance of the nuclear-spin readout and initialization procedure.

The coherence times for both nuclear and electron spins are characterized by means of Ramsey and Hahn-echo measurements (Fig. 1e). For the nuclear spins, the phase coherence time extracted from Ramsey measurements, ${T}_{2}^{\star }$, ranges from 1 to 46 ms. Refocusing with Hahn echo greatly extends such a phase coherence, ${T}_{2}^{{\rm{Hahn}}}$, to values between 3 and 660 ms. We observe that the phase coherence of the data qubits is related to its hyperfine Stark coefficient (Supplementary Information Section VII). Accordingly, we note that deterministic atom placement will provide a way to improve coherence by tailoring the spin registers for smaller susceptibility to electric field fluctuations. For the electrons *e*_1_ and *e*_2_, we measure similar phase coherence times of ${T}_{2}^{\star }\approx 20\,{\rm{\mu }}{\rm{s}}$ and ${T}_{2}^{{\rm{Hahn}}}\approx 350\,{\rm{\mu }}{\rm{s}}$. Overall, our investigations affirm the potential of refocusing techniques to substantially improve the performance of our 11-qubit atom processor.

Single-qubit randomized benchmarking (1Q-RB) reveals that all qubits except *n*_4_ operate with gate fidelities greater than 99.90% and as high as 99.99% for *n*_5_ (see Supplementary Information Section VIII for optimization details). We attribute this excellent performance to long coherence times and minimal frequency drifts in both ESR and NMR (Supplementary Information Sections III and VII). These single-qubit metrics are on par with our recent results using a single spin register^5^, indicating consistency in atomic-scale fabrication.

**Fig. 2:**
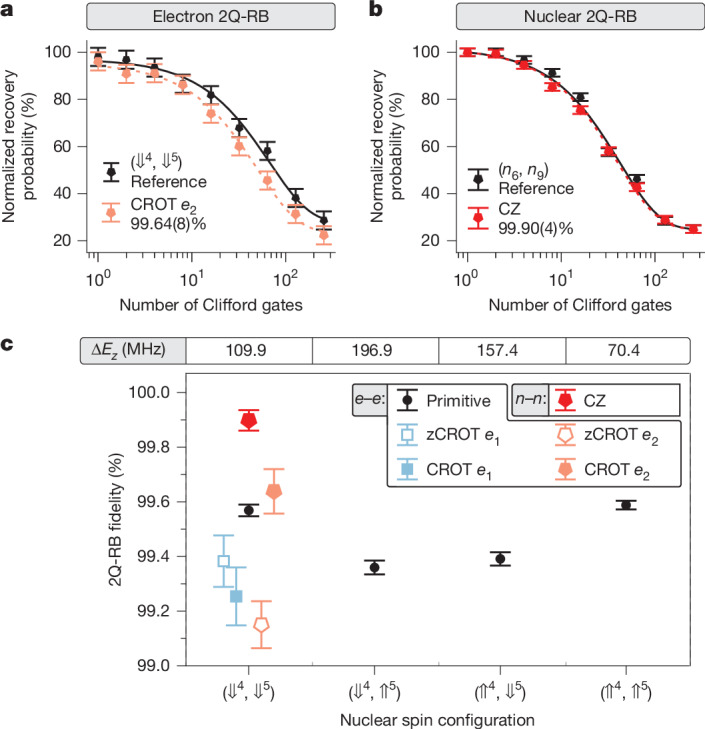
High-fidelity two-qubit operation between nuclear (CZ) and electron (CROT) spins. **a**, Normalized 2Q-RB of the electron–electron CROT gate from the reference (black) and interleaved procedure (CROT *e*_2_) showing the Clifford fidelity. **b**, Normalized 2Q-RB of the geometric CZ operation on the nuclear-spin pair *n*_6_ and *n*_9_ from the reference (black) and interleaved procedure (CZ). All other nuclear spins are initialized to down ⇓ in this experiment. **c**, Summary of the nuclear (CZ) and electron 2Q-RB (zCROT and CROT of *e*_1_ and *e*_2_) fidelities. For the electron CROT gate, the primitive fidelities (reference for interleaved 2Q-RB) are also shown for different nuclear spin configurations, with the corresponding frequency gap Δ*E*_*z*_ = |*f*_CROT e1_ − *f*_CROT e2_| indicated at the top.

To perform multi-qubit operations with any data qubit across our atom processor, we now establish a quantum link between the nuclear spin registers through the exchange interaction of the electrons. We first assess the performance of this link with interleaved two-qubit randomized benchmarking (2Q-RB) of the electron CROT gate (see Methods). To minimize off-resonant population transfer between the zero-controlled rotation (zCROT) and CROT resonances, the Rabi frequency is optimized to *f*_Rabi_ ≈ 400 kHz for the chosen exchange coupling *J* ≈ 1.55 MHz (ref. ^40^) (Supplementary Information Section VIII.C). This choice sets the duration of the CROT π rotation to approximately 1.25 μs. Also, we calibrate the phase offsets of the CROT gates and implement a compensation protocol^30^ (Supplementary Information Section VIII.C). Figure 2a shows the reference and interleaved 2Q-RB data for *e*_2_ when all nuclear spins are initialized to down (⇓⇓⇓⇓, ⇓⇓⇓⇓⇓), which we denote for simplicity as (⇓^4^, ⇓^5^). The extracted electron–electron CROT gate fidelity of 99.64(8)% indicates excellent performance that is relevant for the application of quantum-error-correction protocols.

According to ref. ^35^, the fidelity of the two-qubit CROT gate depends on the Larmor-frequency splitting Δ*E*_*z*_ = |*f*_CROT e1_ − *f*_CROT e2_|, which is defined by the nuclear-spin configuration. In particular, when Δ*E*_*z*_ is similar to the exchange interaction strength *J*, the fidelity is lower owing to hybridization with the singlet–triplet eigenbasis. By choosing small exchange of *J* = 1.55 MHz, we operate at a large Δ*E*_*z*_/*J* ratio and obtain CROT gate fidelities exceeding 99% across different nuclear-spin configurations, as shown in Fig. 2c.

A key task of the ancilla electron in our 14|15 platform is to entangle nuclear data qubits by means of a geometric CZ gate that is implemented through a 2π-ESR rotation^4,5,41^ (for a detailed derivation, see Supplementary Information Section II). Figure 2b shows interleaved 2Q-RB results for the nuclear CZ gate applied on two nuclear spins *n*_6_ and *n*_9_ on the 5P register, giving a nuclear two-qubit-gate fidelity of 99.90(4)%. The nuclear CZ gate strongly outperforms the CROT gate and thus allows local multi-qubit operation on a spin register with high fidelity.

**Fig. 3:**
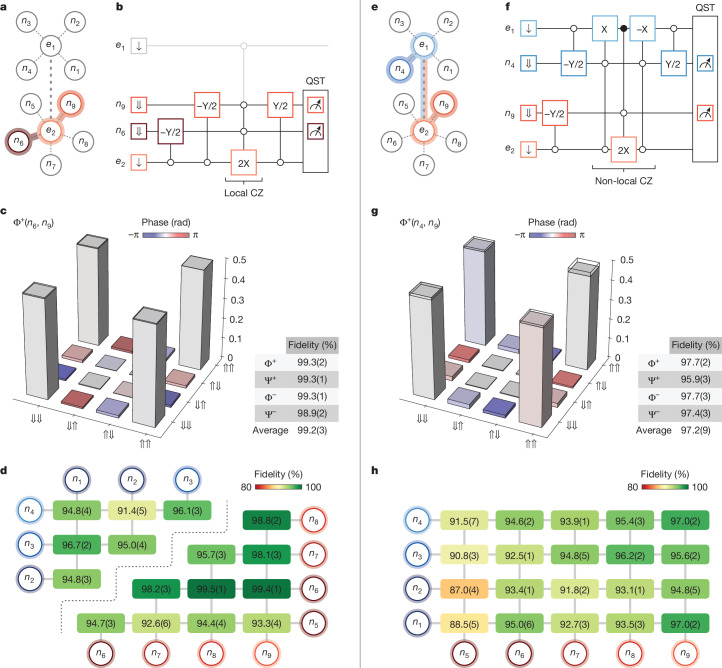
Bell states within a register (left, local) and across registers (right, non-local). **a** (**e**), Connectivity of a local (non-local) Bell state. **b** (**f**), Circuit for generation and measurement of a Φ^+^ Bell state using local (non-local) CZ gate and QST. Open (filled) circles indicate whether the operation is conditional on the down (up) state. **c** (**g**), Reconstructed density matrix for a local (non-local) Φ^+^ Bell state. The table shows the fidelities for all local (non-local) Bell states. Here a complete set of nine projections is used to reconstruct the density matrix. **d** (**h**), Generation fidelities of local (non-local) Φ^+^ Bell state for all combinations of nuclear spins. As we use a reduced set of three projections, small deviations in the generation fidelities occur.

Before applying this electron-exchange-based link to entangle nuclear spins across the two registers, we first benchmark the generation of local Bell states within a single spin register. As an example, we entangle the nuclear spins *n*_6_ and *n*_9_ of the 5P register through the electron *e*_2_ (see schematic in Fig. 3a). An exemplary quantum circuit to prepare the Bell state is shown in Fig. 3b, which uses this nuclear CZ gate to entangle the nuclear-spin pair. Accordingly, the four maximally entangled Bell states, $| {\Phi }^{\pm }\rangle =(| \Downarrow \Downarrow \rangle \pm | \Uparrow \Uparrow \rangle )/\sqrt{2}$ and $| {\Psi }^{\pm }\rangle =(| \Downarrow \Uparrow \rangle \pm | \Uparrow \Downarrow \rangle )/\sqrt{2}$ can be generated by adjusting the phase of the initial −Y/2 NMR pulses, by inverting their respective signs. We remind that the gate operations used are conditional on the spectator data qubits in the system, which are initialized to ⇓. We perform quantum state tomography (QST) using a complete set of nine projections (all combinations of X, Y and Z for the two data qubits) and reconstruct the corresponding density matrix (Methods and Fig. 3c). Here the experiments are performed with *J* ≈ 1.69 MHz, which sets the optimal Rabi frequency for CROT operations to 436 kHz. Without removal of state preparation and measurement (SPAM) errors, we obtain an average state fidelity of 99.2(3)% for all Bell states (see table in Fig. 3c). To characterize the local Φ^+^ state across all nuclear-spin pairs from the two registers, we reconstruct the density matrix from a reduced set of three projections (XX, YY and ZZ). This way, we can increase the measurement speed with similar accuracy^42^ (Supplementary Information Section IX). For nuclear spins with smaller hyperfine coupling than *J*, we reduce *f*_Rabi_ for CROT gates to minimize off-resonance driving (Supplementary Information Section VIII.C). Figure 3d shows the local Φ^+^ state fidelities for all local combinations of data qubits on the respective registers ranging from 91.4(5)% to 99.5(1)%. To the best of our knowledge, the peak Bell-state fidelity surpassing 99% represents the highest value reported in semiconductor devices so far.

The variation in Bell-state fidelities observed arises from the interplay between several effects, including the Stark coefficient, the operational speed, the stability of the qubit frequencies, microwave-induced frequency shifts and the coherence time of the qubits involved (compare Supplementary Information Sections III, VII and VIII). For instance, Bell states involving *n*_5_ exhibit lower fidelities (see the corresponding row in Fig. 3d). This reduction is primarily caused by its small hyperfine coupling, which sets the CROT gate speed approximately three times slower than for the other nuclear spins (*n*_6_–*n*_9_) in the same register (Supplementary Information Section VIII.D).

As a next step, we now interconnect the spin registers and implement non-local Bell states over the electron-exchange-based link. To demonstrate the approach, we entangle nuclear spins *n*_4_ and *n*_9_ through both electrons *e*_1_ and *e*_2_ (see connectivity in Fig. 3e). To implement the non-local CZ gate in the regime in which *J* ≪ Δ*E*_*z*_, we project the targeted nuclear state on the electron *e*_1_ through X gates (π rotation), sandwiching the 2X operation on *e*_2_ (see circuit in Fig. 3f for the example of the Φ^+^ state). Again, we perform QST using a complete set of nine projections to maximize measurement accuracy. Figure 3g shows the density matrix of the non-local Bell state Φ^+^ with a table listing the extracted fidelities for Φ^+^, Φ^−^, Ψ^+^ and Ψ^−^, with an average of 97.2(9)%.

We characterize the non-local Φ^+^ state for all combinations of nuclear-spin pairs across the registers. Figure 3h shows the obtained state fidelities ranging from 87.0(4)% to 97.0(2)%. The observed reduction in fidelity compared with local Bell states is primarily attributed to the increased operation time of the non-local CZ gate. In particular, entanglement involving nuclear spins with smaller hyperfine coupling (*n*_1_, *n*_2_ or *n*_5_) exhibits slightly lower performance, underscoring the importance of engineering hyperfine couplings larger than the exchange strength *J* in future devices. These results demonstrate the ability to generate pairwise entanglement between arbitrary nuclear-spin pairs, highlighting the potential of the 14|15 platform to realize efficient all-to-all connectivity.

**Fig. 4:**
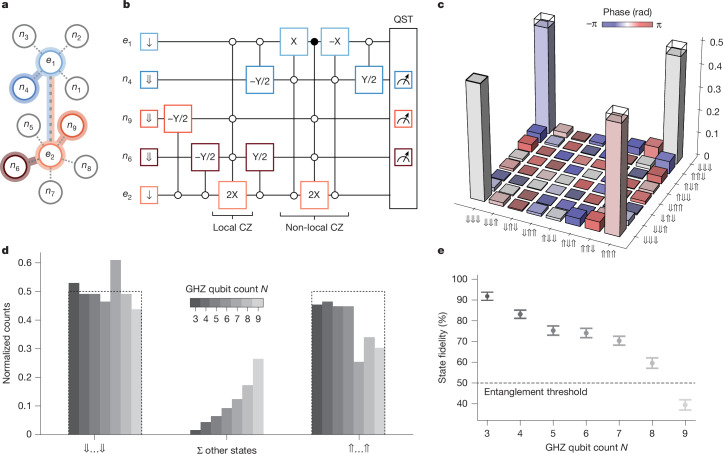
Non-local multi-qubit GHZ states. **a**, Connectivity of the three-qubit GHZ state comprising *n*_4_, *n*_6_ and *n*_9_. **b**, Circuit for the generation and measurement of the three-qubit GHZ state using the local and non-local CZ gate and QST through the ancilla qubits *e*_1_ and *e*_2_. Open (filled) circles indicate whether the operation is conditional on the down (up) state. **c**, Reconstructed density matrix for the GHZ state with *N* = 3 entangled nuclear spins. **d**, Normalized QST counts in the *z* projection—that is, diagonal of density matrix—for GHZ states with increasing qubit count *N*. The bars on the left (right) show matrix elements in which all nuclear spins are down ⇓…⇓ (up ⇑…⇑). All other elements with mixed states (with ⇓ and ⇑) are combined via their sum in the bars in the middle. **e**, Generation fidelity as a function of the number of qubits *N* in the GHZ state.

A straightforward approach to benchmarking the all-to-all connectivity of a quantum processor is the generation of GHZ states with an increasing number of qubits. Accordingly, we investigate in the following non-local multi-qubit entanglement with an increasing number of nuclear spins. First, we generate a GHZ state with three nuclear spins: *n*_4_ on the 4P register and *n*_6_ and *n*_9_ on the 5P register (Fig. 4a). We implement a combination of local and non-local Bell states and concatenate the corresponding entanglement circuits as shown in Fig. 4b. The density matrix shown in Fig. 4c is reconstructed from a full set of QST measurements. Without SPAM removal, we report a GHZ state fidelity of 90.8(3)%.

To prepare a GHZ state with more than three qubits, we now extend the circuit shown in Fig. 4b by adding the local entanglement sequence—NMR  −Y/2, local ESR 2X and NMR Y/2—for each extra qubit. For the 5P (4P) register, we add these local entanglement operations before (after) the non-local CZ. Because the number of tomography bases grows exponentially (3^*N*^, in which *N* is the number of qubits), we use a reduced measurement strategy that requires only *N* + 1 bases to estimate the state fidelity^42,43^. Figure 4d shows the counts in the *z* basis of GHZ states with an increasing number of entangled nuclear spins. In the ideal GHZ state, measurement outcomes are equally distributed between the states in which all nuclear spins are either down (⇓…⇓) or up (⇑…⇑). Increasing the number of qubits in the GHZ state (*N*), we observe a gradual increase in the probability of all other states, that is, mixed combinations of ⇓ and ⇑. The corresponding GHZ fidelities are plotted in Fig. 4e. The three-qubit GHZ fidelity is 92(2)%, consistent with the value of 90.8(3)% obtained from full QST. Because a fidelity greater 50% is sufficient to witness genuine *N*-qubit entanglement^44^, the data demonstrate that entanglement is maintained for up to eight nuclear spins. Further performance improvements are anticipated by coherent control optimization^45^, frequency crosstalk mitigation and the incorporation of refocusing pulses. Building on this progress, the present results demonstrate efficient connectivity across nuclear data qubits in our atom processor, representing an important step towards future implementations of quantum error correction on the 14|15 platform.

## Conclusion

By coupling a 4P and a 5P register by means of electron exchange interaction, we considerably exceeded the number of interconnected qubits with respect to previous works in semiconductor devices^4,5,16–19^ and achieve an important milestone towards a modular spin qubit system within the 14|15 platform. While increasing the number of connected qubits, we have shown that physical-level benchmarks are maintained and some of them even improved, with two-qubit gate fidelities reaching 99.9% for the first time in silicon qubits. Systematic characterization of the 11-qubit atom processor enabled the development of tailored calibration routines that scale linearly with more registers. By using the electron spin on each of the two registers as an ancilla qubit, we implemented efficient single-qubit and multi-qubit control for all nuclear spins. This level of performance has allowed us to entangle every nuclear-spin pair within the 11-qubit system with Bell-state fidelities ranging from 91.4(5)% to 99.5(1)% within registers and from 87.0(4)% to 97.0(2)% across registers. We expanded the connectivity by preparing multi-qubit GHZ states across all data qubits and showed that entanglement is preserved for up to eight nuclear spins. By successfully introducing a coherent link across spin registers while maintaining excellent qubit performance, we demonstrate a key capability for future implementations in the 14|15 platform aimed at quantum error correction.

In the present work, gate operations are performed under the assumption that spectator qubits remain in a pre-initialized state. Future work will focus on benchmarking performance with arbitrary spectator qubit states^46^, including characterization of error and leakage channels using modified randomized benchmarking protocols^47–49^, gate-set tomography^50^ and non-Markovian process tomography^51^. As implementing a universal geometric CZ gate requires driving all ESR transitions conditional on both ⇑ and ⇓ states of the spectator nuclear spins, we will pursue control optimization through pulse shaping and parallelized drive execution^45^, alongside refined calibration strategies to mitigate microwave-induced frequency shifts^52^. Finally, as small hyperfine couplings limit gate speed, we aim to atomically engineer the registers to optimize hyperfine couplings in future processors^53^.

## Methods

### Experimental set-up

A single-electron transistor serves as charge reservoir and sensor enabling spin readout of the electrons through spin-to-charge conversion. Details of the basic operation of our atom processor are provided in Supplementary Information Section I. The encapsulation is about 45 nm. On top of the chip, an antenna is horizontally offset from the dots by  about 300 nm (refs. ^5,55^). It allows us to drive NMR and ESR. The experiment is performed in a cryogen-free dilution refrigerator at a base temperature of about 16 mK. Spin polarization is activated by a magnetic field *B* ≈ 1.39 T along the [110] crystal direction.

### Randomized benchmarking

For 1Q-RB, we generate ten variations of a random set of Cliffords up to 1,024 gates. Each Clifford gate is chosen from the one-qubit Clifford group containing 24 elements. Using the Euler decomposition, we translate each Clifford to a single native Y(*θ*) rotation sandwiched between two virtual Z(*θ*) gates. Because the latter operation is instantaneous owing to a change of reference frame, the average number of primitive gates per Clifford is exactly one. For each Clifford set, we take 200 (50) single-shot measurements for the electron (nuclei). We perform qubit frequency recalibrations every 12 runs (equivalent to a few minutes time intervals). We measure recovery probabilities *F*_*↑*_(*n*) and *F*_*↓*_(*n*) to both up and down states and fit the data points with *F*(*n*) = *F*_*↑*_(*n*) − *F*_*↓*_(*n*) with *F*(*n*) = *Ap*^*n*^, in which *n* is the sequence length, *A* is the factor containing SPAM errors and *p* is the depolarizing strength. The Clifford gate fidelity *F*_C_, and hence the primitive gate fidelity *F*_P_, is then extracted as *F*_C_ = *F*_P_ = (1 + *p*)/2. In all randomized benchmarking experiments, we calculate the error bars by bootstrapping resampling methods assuming a multinomial distribution^30,40^.

Similarly, for 2Q-RB, we typically generate 20 variations of a random set of Cliffords up to 256 gates. Each Clifford gate is chosen from the two-qubit Clifford group containing 11,520 elements^56^. For the electron, we use the decomposition to CROT rotations as in ref. ^40^, in which the average number of primitive gates, $\bar{n}$, is 2.57. For nuclear spins, the native operations consist of a combination of π/2 NMR pulses for single-qubit rotations and 2π ESR pulses as CZ gates^5^. Similar to 1Q-RB, we measure recovery probabilities to both *↑↑* and *↓↓* to account for SPAM errors. To extract the polarizing strength, we fit *F*(*n*) = *F*_*↑↑*_(*n*) − *F*_*↓↓*_(*n*) with *F*(*n*) = *Ap*^*n*^ as before. The corresponding Clifford and primitive gate fidelities are *F*_C_ = (1 + 3*p*)/4 and ${F}_{{\rm{P}}}=1-(1-{F}_{{\rm{C}}})/\bar{n}$, respectively.

For the interleaved 2Q-RB, we insert the target Clifford after each random gate, effectively doubling the sequence length. We measure the recovery probabilities in the same manner as standard 2Q-RB and extract the interleaved polarizing strength *p*_i_. Accordingly, we extract the interleaved gate fidelity using *F*_i_ = (1 + 3*p*_i_/*p*)/4. The standard deviation is calculated using the same bootstrapping resampling method and standard error propagation analysis.

### Quantum state tomography

To perform QST measurements, we add projection pulses for each qubit to the target basis {*x*, *y*, *z*} before readout. In particular, we apply  −Y/2 (X/2) to project on *x* (*y*). Because *z* is our native basis, there is no need for any extra rotations.

For Bell-state and GHZ-state generation, we merge the projection pulse with the last Y/2 rotation. Accordingly, when projecting to *x*, the rotations cancel each other and thus we remove them both. For projections to *y*, we convert the sequence Y/2 + X/2 into  −Z/2 + Y/2 according to the Euler decomposition, as the virtual Z rotation, which is implemented by a global phase shift, does not require a physical pulse.

The full QST is taken by projecting to all 3^*N*^ basis, in which *N* is the number of qubits involved. We perform 2,000 single-shot measurements per basis and apply post-selection to ensure successful nuclear spin initialization. The density matrix is reconstructed by performing a constrained Gaussian linear least-squares fit to the tomography counts. The standard deviation is then extracted from Monte Carlo bootstrapping resampling^5,40,57^.

## Online content

Any methods, additional references, Nature Portfolio reporting summaries, source data, extended data, supplementary information, acknowledgements, peer review information; details of author contributions and competing interests; and statements of data and code availability are available at 10.1038/s41586-025-09827-w.

## Supplementary information

Supplementary Information File

## Data Availability

The raw data used in this article are available from Zenodo at 10.5281/zenodo.15549984 (ref. ^58^).

## Code availability

The code used to analyse the data and produce the figures in this article is available from Zenodo at 10.5281/zenodo.15549984 (ref. ^58^).
