# Supplementary material for: An 11-qubit atom processor in silicon
Source: Nature. 2025 Dec 17;648(8094):569–75. doi: 10.1038/s41586-025-09827-w (PMC12711558; doi:10.1038/s41586-025-09827-w)
Supplement: Supplementary file 1 — Supplementary Information File [file 41586_2025_9827_MOESM1_ESM.pdf]

nature portfolio

https://doi.org/10.1038/s41586-025-09827-w

Supplementary information

An 11-qubit atom processor in silicon

In the format provided by the authors and unedited

SUPPLEMENTARY MATERIALS
An 11-qubit atom processor in silicon

Hermann Edlbauer,[1,2] Junliang Wang,[1,2] A. M. Saffat-Ee Huq,[1] Ian Thorvaldson,[1] Michael T. Jones,[1] Saiful Haque Misha,[1] William J. Pappas,[1] Christian M. Moehle,[1] Yu-Ling Hsueh,[1] Henric Bornemann,[1] Samuel K. Gorman,[1] Yousun Chung,[1] Joris G. Keizer,[1] Ludwik Kranz,[1,3] and Michelle Y. Simmons[1,3,4]

[1]*Silicon Quantum Computing Pty Ltd, UNSW Sydney, Sydney, New South Wales, Australia*

[2]These authors contributed equally to this work.

[3]These authors jointly supervised this work.

[4]Corresponding author: michelle.simmons@sqc.com.au

CONTENTS

I. Basic operation of the 11-qubit atom processor 1

II. Physical description of CROT and geometric CZ gates 1

III. Characterisation and recalibration of electron-spin resonances (ESR) 3
- A. Frequency stability of ESR 3
- B. ESR-calibration protocol for multi-nuclear spin registers 3
- C. Frequency stability of electron-exchange coupling J 5

IV. Quantum-non-demolition (QND) nuclear-spin readout 6

V. Optimisation of nuclear spin initialisation 7

VI. Nuclear State Preparation And Measurement (SPAM) analysis 8

VII. Frequency stability of nuclear magnetic resonances (NMR) 9

VIII. Optimisation of gate operations 12
- A. Compensation of frequency shifts induced by microwave drive 12
- B. Optimisation of NMR Rabi frequencies 14
- C. Optimisation of the electron-exchange-based CROT operation 16
- D. Optimal CROT gate speed for CZ operation 18

IX. Bell-state tomography with reduced projections 18

References 19

I. BASIC OPERATION OF THE 11-QUBIT ATOM PROCESSOR

We operate the spin registers via two gates (gate 1 and 2), a single-electron transistor (SET) and an on-chip antenna. Figure S1a shows the hydrogen lithography mask for the gates and the SET after incorporation of the spin registers. Besides its reservoir functionality, the SET serves as charge-sensor via a radio-frequency (RF) reflectometry setup enabling ramped single-shot spin readout [1]. Gate 1 and 2 control the number of electrons on each register that are loaded from a single-electron transistor (SET) located 17(1) nm away from the registers (center to edge). Figure S1b shows the SET signal (in arbitrary units) measured over the (1,1) charge region where one electron is loaded on each of the spin registers. To initialise and readout the spins of the electrons, we move the system to the breaks of the Coulomb peaks indicated as e_1 and e_2. Between spin initialisation and readout, we bring the gate voltages into an intermediate position where we drive nuclear-magnetic-resonance (NMR) and electron-spin-resonance (ESR) via a broadband antenna, which is located on top of the chip at a horizontal distance of ≈300 nm [2, 3].

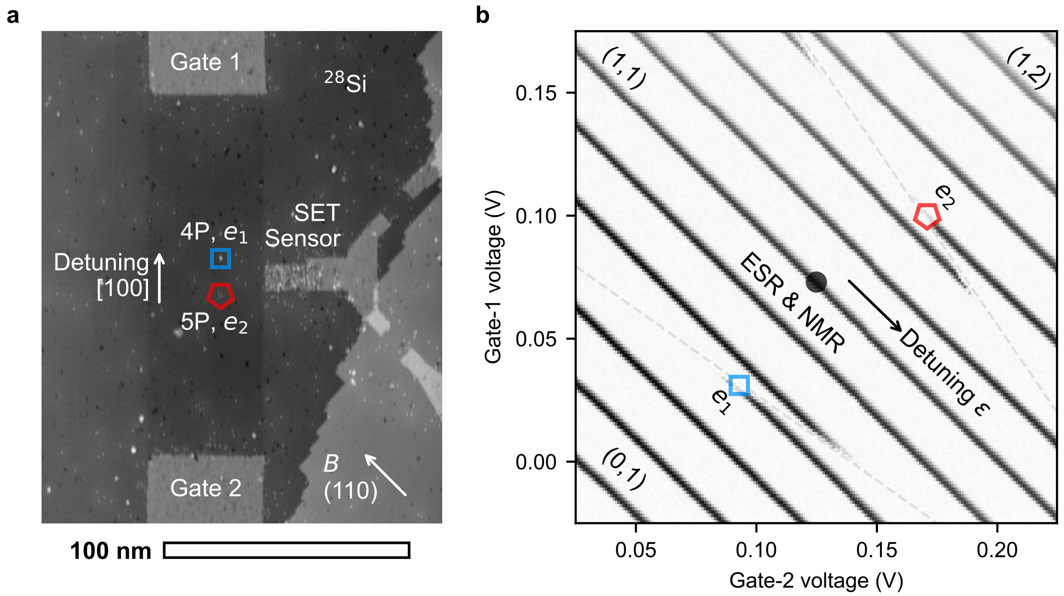

FIG. S1. **Basic operation of the 11-qubit atom processor via detuning gates. a,** Scanning tunneling micrograph showing the core of the atom processor with the detuning gates 1 and 2 and the single electron transistor (SET). The antenna that drives ESR and NMR is located on top of the silicon chip. **b,** Gatemap showing the antenna-operation point (ESR & NMR) and working points for initialisation and readout of the electrons (e_1 and e_2). The grey, dashed lines indicate the separation between the charge configurations (N,M) with integer numbers N and M indicating the number of electrons in the 4P and 5P register. The arrow indicates the direction of voltage detuning ε of gate 1 and 2 to shift the antenna-operation point to configurations with stronger electron exchange.

II. PHYSICAL DESCRIPTION OF CROT AND GEOMETRIC CZ GATES

The spin Hamiltonian of a system with n electrons and m ^{31}P nuclei can be generally described as

$$H = \gamma_e \boldsymbol{B} \cdot \sum_{i=1}^{n} \boldsymbol{S}_i + \gamma_n \boldsymbol{B} \cdot \sum_{j=1}^{m} \boldsymbol{I}_j + \sum_{i=1}^{n} \sum_{j=1}^{m} A_{ij} \boldsymbol{S}_i \cdot \boldsymbol{I}_j + \sum_{i=1}^{n-1} \sum_{j>i} J_{ij} \boldsymbol{S}_i \cdot \boldsymbol{S}_j, \tag{1}$$

where $\boldsymbol{B}$ is the external magnetic field, $\boldsymbol{S}$ is the electron spin Pauli operator and $\boldsymbol{I}$ is the nuclear spin Pauli operator. The first term is the electron Zeeman with gyromagnetic ratio γ_e (which can vary depending on the electron g factor). The second term is the ^{31}P nuclear Zeeman with gyromagnetic ratio $\gamma_n \approx -17.23$ MHz/T. The third term is the hyperfine interaction between the electron spins and ^{31}P nuclear spins, characterised by the hyperfine coupling A. The final term is the exchange interaction between the electron spins, characterised by the exchange coupling J. In our system there are a total of two electrons and nine nuclei ($n = 2$ and $m = 9$), with one electron and 4/5 nuclear spins

on each dot. The hyperfine interactions between electron spin with the nuclear spins on the other dot are negligible. Accordingly, the Hamiltonian for the present 11-qubit atom processor is:

$$H = \gamma_e \boldsymbol{B} \sum_{i=1}^{2} \boldsymbol{S}_i + \gamma_n \boldsymbol{B} \cdot \sum_{j=1}^{9} \boldsymbol{I}_j + \sum_{j=1}^{4} A_j \boldsymbol{S}_1 \cdot \boldsymbol{I}_j + \sum_{j=5}^{9} A_j \boldsymbol{S}_2 \cdot \boldsymbol{I}_j + J \boldsymbol{S}_1 \cdot \boldsymbol{S}_2, \tag{2}$$

The controlled-rotation (CROT) gate for the electron spin is based on the exchange interaction J. Solving the 4×4 spin Hamiltonian with two electrons in the small exchange regime ($J \ll \Delta B_z$, where ΔB_z is the difference in the total hyperfine between the two dots) gives four energy levels: $|\uparrow\uparrow\rangle$, $|\uparrow\downarrow\rangle$, $|\downarrow\uparrow\rangle$, $|\downarrow\downarrow\rangle$. In particular, the CROT (zCROT) gate rotates the target electron spin conditional to the $\uparrow$ ($\downarrow$) state of the control electron spin.

For the nuclear CZ gate, we rely on geometric phase kickback of controlled rotations [3–5]. Specifically, when a spin-1/2 qubit is rotated by an angle of 2π around the Bloch sphere, the unitary implemented on the the state of the system is not the identity I, but instead $-I$, requiring a full 4π rotation to implement I. This is because $e^{-i\sigma_x \theta/2}$, the unitary of an x-axis rotation by θ, evaluates to $-I$ when $\theta = 2\pi$. For single-qubit operations, this manifests as a global phase and can therefore be ignored. However, a conditional 2π rotation introduces a relative phase, enabling the implementation of a multi-qubit gate.

For example, consider an ESR 2π rotation of the electron e_1, conditional on the register's spin state $|\Uparrow\Uparrow\Uparrow\Uparrow\rangle$. The total unitary in this case would be given by:

$$U = |\Uparrow\Uparrow\Uparrow\Uparrow\rangle \langle\Uparrow\Uparrow\Uparrow\Uparrow| \otimes (-I) + \left[I^{\otimes 4} - |\Uparrow\Uparrow\Uparrow\Uparrow\rangle \langle\Uparrow\Uparrow\Uparrow\Uparrow|\right] \otimes I \tag{3}$$

$$U = \Big(\left[I^{\otimes 4} - |\Uparrow\Uparrow\Uparrow\Uparrow\rangle \langle\Uparrow\Uparrow\Uparrow\Uparrow|\right] - |\Uparrow\Uparrow\Uparrow\Uparrow\rangle \langle\Uparrow\Uparrow\Uparrow\Uparrow| \Big) \otimes I \tag{4}$$

This means that the electron experiences $-I$ if the nuclei are $|\Uparrow\Uparrow\Uparrow\Uparrow\rangle$, and I otherwise. The unitary given in Eq. 4, ignoring the identity to the electron, is exactly the unitary of a $\mathrm{C}^3\mathrm{Z}$ on the register, *i.e.*, a controlled-Z gate on three other qubits. Similarly, any state addressable via ESR can be marked with a such a -1 phase. As an example, if the spectator nuclear spins are initialised and uninvolved in the computation (*e.g.*, in the generation of a Bell state pair), then a single ESR 2π rotation is a direct two-qubit CZ gate. Otherwise, a two-qubit CZ gate can be performed using multiple $\mathrm{C}^n\mathrm{Z}$ gates [3] either sequentially or in parallel. Here, $n = m - 1$ where m is the number of nuclear spins in the register. This establishes the mechanism to perform nuclear CZ gates, along with general $\mathrm{C}^n\mathrm{Z}$ gates locally within a single register.

When performing non-local gates between multiple registers, we leverage the fact that ESR transitions are also conditional on the electron state due to the exchange-interaction (J) term of Eq. 2. This allows ESR on one register to not only be conditioned on the local nuclear spins, but also on the electron state ($|\uparrow\rangle / |\downarrow\rangle$) of the exchange-coupled register. By first applying a π ESR rotation the electron of the local register conditional on its nuclear spin configuration, and then performing a 2π ESR rotation on the exchange-coupled electron, the geometric phase can be made to be conditional on the combined nuclear configuration of both registers. The combined circuit to implement an inter-register ("non-local") nuclear gate is therefore:

1. Perform a π ESR rotation on e_1 conditional on its register spin state.
2. Perform a 2π ESR rotation on the exchange-coupled electron e_2 conditional on both e_1 and the corresponding register spin configuration.
3. Perform a $-\pi$ ESR rotation on e_1 to undo the initial operation.

Combined, this process will mark a two-register nuclear state with a phase of -1, similar to the local $\mathrm{C}^n\mathrm{Z}$ gate. Note that it is important that the last step uses a $-\pi$ rotation rather than π to avoid an unwanted geometric phase gate occurring locally on the first register. This circuit is illustrated in main text Fig. 4b.

We remark that this process produces a $\mathrm{C}^n\mathrm{Z}$ gate. Therefore, same strategies for local $\mathrm{C}^n\mathrm{Z}$ gates can be employed to produce two-qubit gates between registers. In addition, the inter-register gate can be made to perform anti-controlled operations similarly to the local version. We highlight that this inter-register gate is optimised for multi-qubit registers, and it is therefore different to that presented by Madzik *et al.* [6], despite also being mediated by electron exchange.

III. CHARACTERISATION AND RECALIBRATION OF ELECTRON-SPIN RESONANCES (ESR)

A. Frequency stability of ESR

To track the resonant ESR frequencies conditional on a given reference and target nuclear spin state, we employ the quantum circuit shown in Fig. S2a. The schematic shows the protocol by the example of electron e_1 and a subsystem of corresponding nuclear spins n_1 and n_2. To minimise the chance for frequency jumps or drifts, we interleave ESR measurements for a reference and target state. Prior to the reference measurement we initialise all electron and nuclear spins of the system to the down state ($\downarrow\Downarrow^4$,$\downarrow\Downarrow^5$). After the reference measurement, we transition into the target state via a set of NMR π rotations (X gates) on the specific nuclear spins—that is only a single X gate on n_2 in the present example. High-precision ESR measurements are achieved by coherent ESR drive of n periods of π (X^n). Here we have chosen $n = 9$ rotations to balance accuracy versus measurement duration. As we perform a fine frequency sweep of the two signals around the reference (f_0) and target (f_1) frequency, we obtain two precise spectra and thus the ESR offset.

Investigating the ESR stability, we find different results for the 4P and 5P register: Figure S2b shows time evolutions of the ESR frequencies $f_{\mathrm{ref.}}$ of each register conditional to all nuclear spins being in the down-state ($\Downarrow^4$,$\Downarrow^5$). The corresponding histograms are shown in Fig. S2c. Remarkably, the 4P register is stable for the entire tracking period (up to 15 hours) with a standard deviation $\sigma = 0.90(5)$ kHz. For the 5P register, on the other hand, we observe distinct frequencies indicating the presence of two-level systems (TLS) with an extent of 10 kHz and 45 kHz. Since these jumps are only observed for one register, we rule out the possibility of electrostatic fluctuations which should affect both registers equally. We performed ionised NMR experiments [7], and observed no signal from Si^{29}. For the fidelity of qubit manipulations, the broadening of the Rabi frequency $f_{\mathrm{Rabi}} \approx 430$ kHz renders this 10 kHz shift negligible. The 45-kHz jumps are less frequent and occur over tens of minutes or longer. By performing recalibration every 5 to 10 minutes for sensitive experiments such as Bell State tomography, we are able to mitigate the effect of these slow fluctuations. Based on these results, we speculate that the stability of the ESR frequency is either determined by the specific geometry of the nuclear spin registers or growth imperfections. Accordingly, we anticipate an increased ESR stability with further advances in deterministic-incorporation techniques.

B. ESR-calibration protocol for multi-nuclear spin registers

The presence of any TLS would require frequent recalibration of ESR frequencies. Since the number of ESR frequencies is 2^k for each multi-nuclear spin register, where k is the number phosphorus atoms, there are in total 48 ESR (2^4+2^5) frequencies to keep track of when the electrons are initialised to $\downarrow$. A typical recalibration measurement for a single frequency takes approximately 1 minute. Consequently, performing a full calibration of the system could take up to 50 minutes. Such a procedure is impractical, as the earliest calibrated frequencies may already have drifted by the time the process is complete.

To overcome this challenge, we analyze the correlations in the ESR fluctuations for different nuclear spin-configurations. Figure S2d and S2e show the statistical distribution of the ESR frequency offset for all nuclear spin configurations referenced to the ($\Downarrow^4$,$\Downarrow^5$) ESR. Since the fluctuations are similar for all states, we find that the ESR frequencies shift collectively. The mean standard deviation of the 4P register, $\bar{\sigma}_{\mathrm{4P}} \approx 0.8$ kHz, is consistent with the extracted deviation of 1 kHz from tracking $f_{\mathrm{ref.}}$. For the 5P system, all frequency offsets exhibit deviations less than 10 kHz. Since $\bar{\sigma}_{\mathrm{5P}} \approx 1.5$ kHz is much lower than 45 kHz which is the extent of the largest fluctuation on the 5P register (compare Fig. S2bc), the TLS jumps occur simultaneously for all ESR resonances. Therefore, by calibrating the reference frequency $f_{\mathrm{ref.}}$ we can update all drive frequencies employing the characterised frequency offsets—proving a scalable ESR-recalibration protocol for multi-nuclear spin registers.

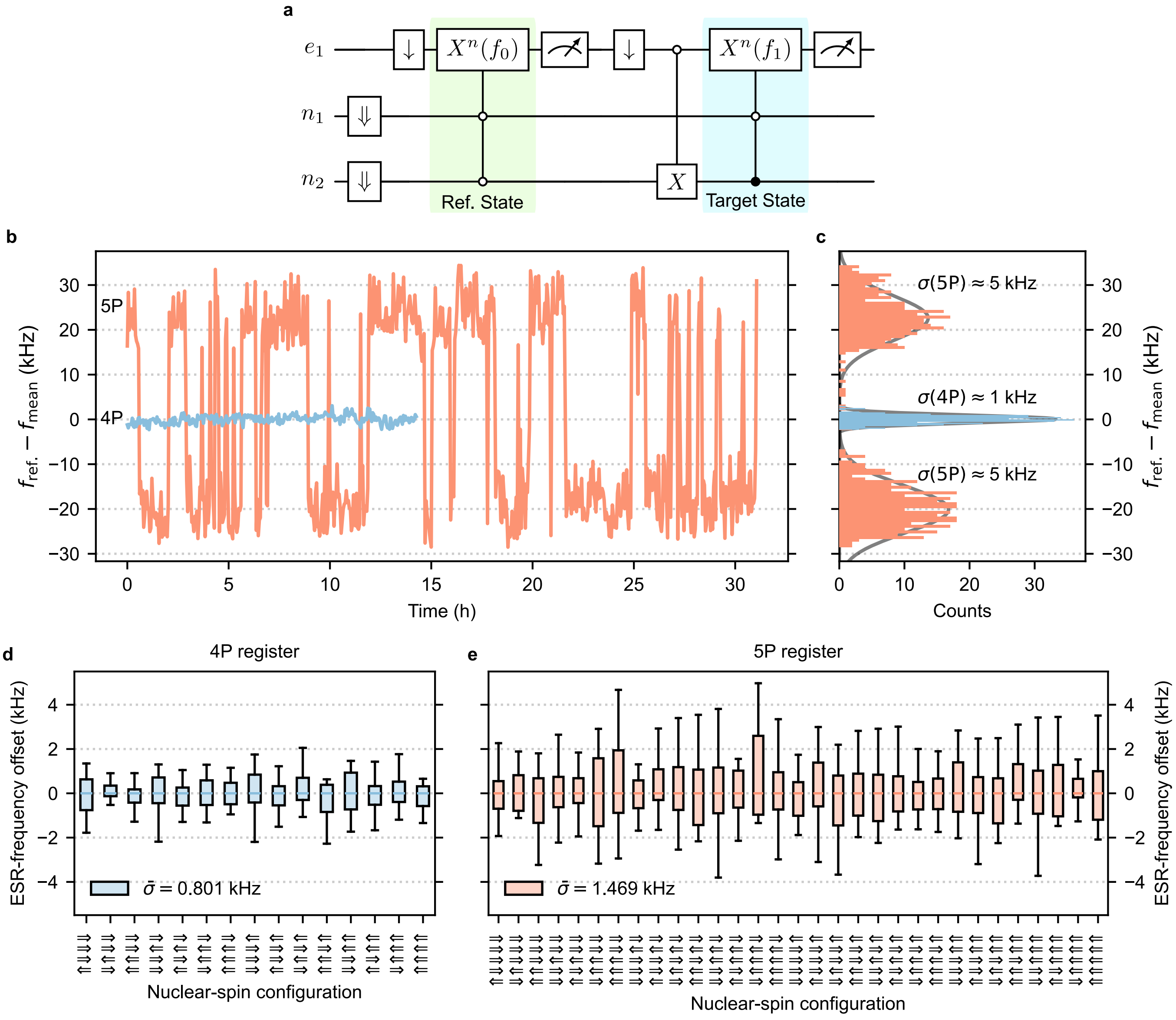

FIG. S2. **Stability of the ESR frequencies over time.** **a,** Circuit diagram for tracking the relative offset between an ESR transition for a target nuclear spin and a reference nuclear spin state. X^n indicates the number n of π rotations on the electron. f_0 is the frequency parameter to scan across the reference ESR peaks ($f_{\mathrm{ref.}}$) where all nuclear spins are in the down state ($\Downarrow^4$,$\Downarrow^5$). f_1 is the frequency parameter to measure a targeted ESR peak (f_{target}) in any other nuclear configuration. f_0 and f_1 are swept in parallel to extract the resonant ESR frequencies $f_{\mathrm{ref.}}$ and f_{target} relative to each other. **b,** Evolution of the reference ESR transition in which all nuclear spins $\Downarrow$ for 4P (blue) and 5P (orange) over several hours. To compare the traces from the 4P and 5P register, we subtract f_{mean} from the data. Here, $n = 9$ rotations are employed. **c,** Histograms of $f_{\mathrm{ref.}}$ from **b**. **d (e),** Boxplots of the frequency offset $f_{\mathrm{target}} - f_{\mathrm{ref.}}$ distribution for all possible nuclear spin states in the 4P (5P) register with the mean standard deviation, $\bar{\sigma}$. For comparison, the values are centered at the median value.

C. Frequency stability of electron-exchange coupling J

The stability measurement of the exchange interaction J is similar to the protocol shown in Fig. S2a. In the reference measurement, we have all spins initialised to the down state: $(\downarrow\Downarrow^4, \downarrow\Downarrow^5)$. By having the control-electron in the $\downarrow$ state, the corresponding ESR measurement is thus probing the zCROT branch. To measure electron-exchange coupling, the target measurement is modified accordingly: Instead of changing the nuclear configuration, we invert the control electron to the spin-up-state $\uparrow$ via an ESR π pulse and thus target the corresponding CROT branch. As we repeatedly measure the frequency gap between the zCROT and CROT branches, we can characterise the fluctuations of the electron-exchange coupling J. Figure S3 shows the results of such stability measurement for the electrons e_1 and e_2 on the 4P and 5P registers. Over 10 hours, the fluctuations of J remain below 1.8 kHz, indicating that any TLS jumps do not affect the exchange interaction. Accordingly, we can simply recalibrate zCROT only and assign the drive frequency for CROT using the frequency gap ($J = 1.690$ MHz in this configuration). Compared to the full calibration protocol, we thus half the number of recalibration measurements for electron two-qubit-gate operations.

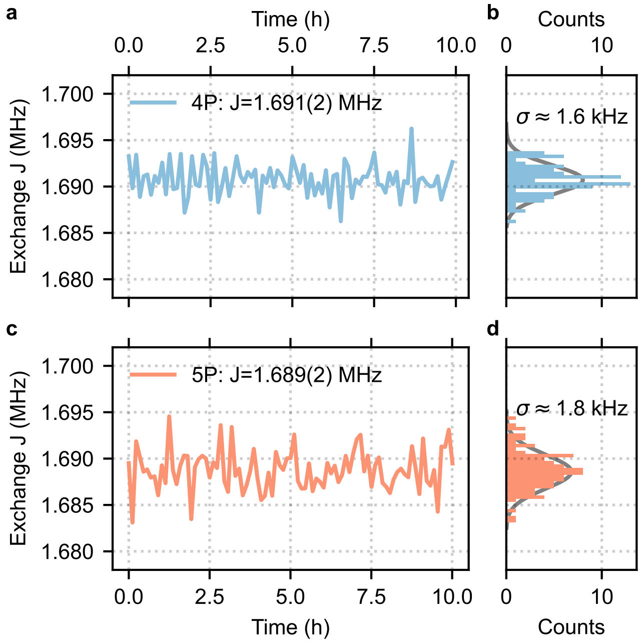

FIG. S3. **Stability of the exchange interaction, J. a (c),** Evolution of the exchange strength $J = f_{\mathrm{CROT}} - f_{\mathrm{zCROT}}$ from 4P (5P) over 10 hours. The employed quantum circuit is a modified version of S2a where the reference and target transitions are the zCROT and CROT operations, respectively. Here, all nuclear spins are initialised to $\Downarrow$. **b (d),** Histogram of J from **a** (**c**).

IV. QUANTUM-NON-DEMOLITION (QND) NUCLEAR-SPIN READOUT

To measure the state of the nuclear spin, we perform quantum non-demolition (QND) readout [3, 8]. Using the electron spin as an ancilla qubit, we map the target nuclear spin state to the electron spin via a sequence of conditional ESR pulses. Figure S4a shows an example of a QND-readout circuit for a subsystem of n_1 and n_2 of the 4P register. To account for variations in electron-spin readout visibility, we perform two sequences of 2^{k-1} π-ESR rotations (X gate) that are conditional on $n_1 =\Downarrow$ and $n_1 =\Uparrow$ and unconditional on the other nuclear spins. Here, k is the total number of nuclear spins in the register. In the present example, $k = 2$ with n_2 being the only other nuclear spin considered. As the ESR drives are sandwiched by an electron $\downarrow$-initialisation and readout, we project the nuclear spin on the electron state. Repeating the cycle N times we obtain the probabilities ($P_\Uparrow$ and $P_\Downarrow$) of the nuclear spin to be in the up or down state. Then, we classify the nuclear spin being $\Uparrow$ as $\Delta P = P_\Uparrow - P_\Downarrow \geq 0$.

In contrast to previous works [3], we use fast coherent rotations instead of slow adiabatic inversion pulses to drive the ESR transitions. In this way, we can speed-up readout cycles and reduce the probability of nuclear spin-flip events during the QND readout. A prerequisite to implementing such coherent drive process is to have all ESR resonance frequencies well calibrated —see Supplementary Material III.

To characterise nuclear-spin readout, we perform 1000 repetitions of the circuit with $N = 100$ read shots. An exemplary histogram of ΔP from such an experiment is shown in Fig. S4b. By fitting the two distinct peaks with a

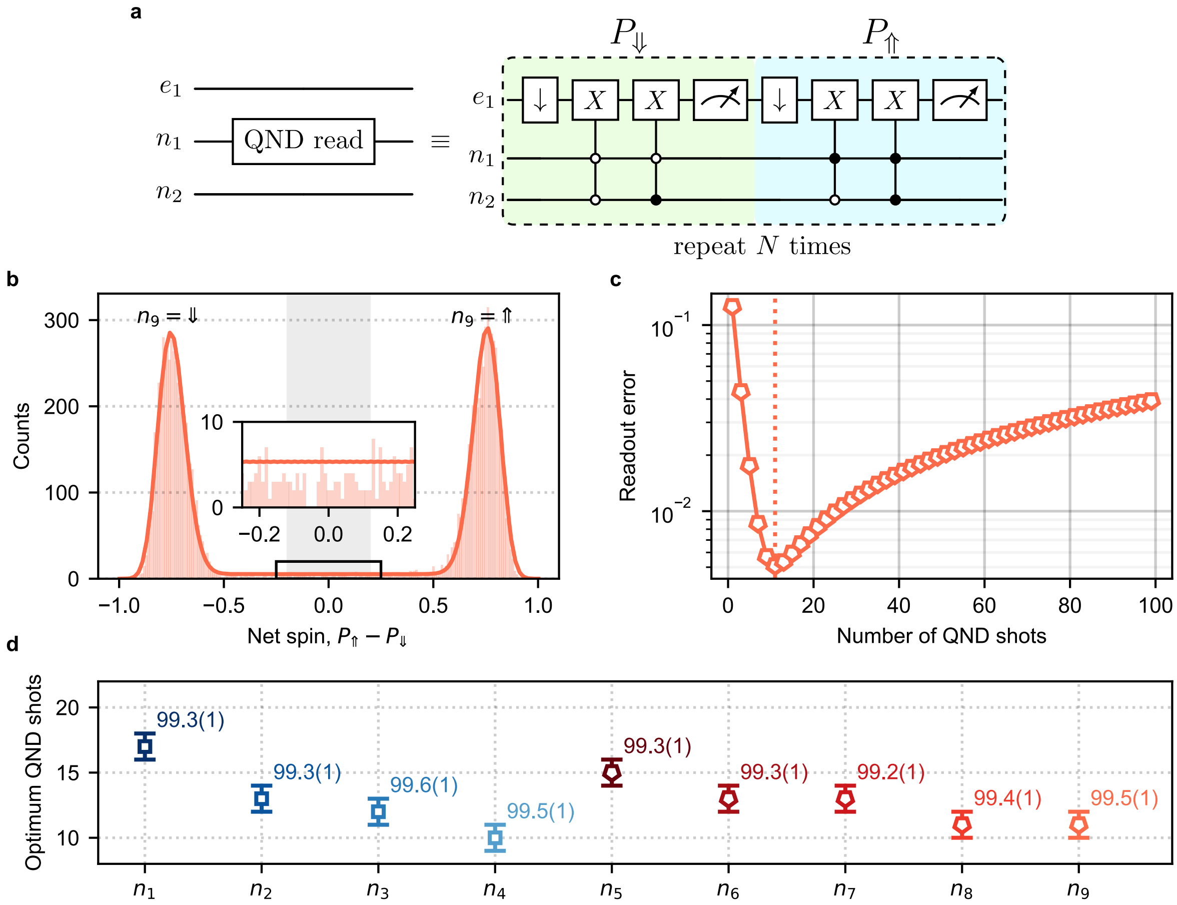

FIG. S4. **Quantum-non-demolition (QND) readout of the nuclear spins. a,** Exemplary QND-readout circuit of nuclear spin n_1 in the reduced subsystem with dependency on n_2 only. **b,** Histogram of the net spin, $P_\Uparrow - P_\Downarrow$, from $N = 100$ QND shots for qubit n_9. The solid line is the fit using a Markov chain model. The grey shaded region (typically, ± 0.2) highlights the range at which net spin values are rejected in post-selection to remove uncertain spin classifications. The inset shows the histogram baseline. **c,** The readout error extracted from **b** where the dotted line indicates the optimum number of QND shots for highest readout fidelity. **d,** Optimum number of QND shots for all nuclear spins and their corresponding readout fidelity in percent (%).

Markov-chain model [3], we reproduce the finite background (inset), which is caused by nuclear spin-flip events during the QND readout cycles, likely due to ionisation shock [8, 9]. We extract spin-flip probabilities per readout cycle of 0.08(2)% and 0.09(1)% for the 4P and 5P registers, respectively. Setting the threshold range appropriately (grey region), we can assess the nuclear-spin-readout fidelity without bias from spin-flip events. Employing the Markov-chain model, we extract the readout error as a function of the number of QND shots as shown in Fig. S4c. Despite an electron-spin readout fidelity of ≈75%, we can increase the number of QND shots to reduce nuclear-spin-readout error. Beyond a certain point, the likelihood of nuclear spin flips becomes significant, leading to a degradation in overall fidelity. The optimum number of shots for QND readout of each nuclear spin is shown in Fig. S4d. Remarkably, we find that we can achieve nuclear-spin-readout fidelity above 99% for all data qubits.

The protocol to readout a specific set of states for all the nuclear spins is almost identical to the circuit that is required to obtain a single, specific nuclear spin state as shown in Fig. S4a. Below, we show a similar example for a readout circuit of both n_1 and n_2 with the target state being $\Downarrow\Downarrow$:

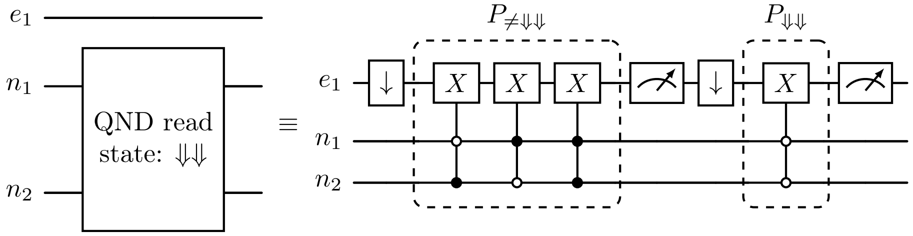

The first half of the circuit ($P_{\neq\Downarrow\Downarrow}$) maps all the nuclear states that are different to the target spin state $\Downarrow\Downarrow$ to the electron spin. Subsequently, we realise the mapping of the target state and calculate the equivalent net value $P_{\text{net}} = P_{\Downarrow\Downarrow} - P_{\neq\Downarrow\Downarrow}$ We then assign the nuclear state as $P_{\text{net}} \geq 0$. Note that this protocol offers a speed advantage over sequential nuclear-spin readout, since it only extracts a single binary outcome indicating if all nuclear spins in the register occupy a specific state. In short, instead of performing QND readout measurements for each of the k nuclear spins, only one sequence is executed.

V. OPTIMISATION OF NUCLEAR SPIN INITIALISATION

The ability to initialise all nuclear spins from a random state to a certain initial state (such as all $\Downarrow$) is essential for the operation of our quantum processor. We follow a protocol used in previous works [3] based on a process called electron state transfer (EST) [4], followed by a verification QND nuclear-state readout to increase initialisation fidelity by post-selecting on the desired target state. An example of an EST circuit initializing n_1 in the subsystem with n_2 is shown below:

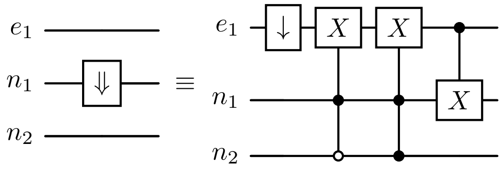

The central idea is to rotate the nuclear spin conditional on the electron spin. For instance, to initialise a given nuclear spin n_1 to $\Downarrow$, we first prepare the electron spin in the $\downarrow$ state with high-fidelity. Subsequently, we rotate the electron to $\uparrow$ using ESR conditional to $|n_1\rangle = |\Uparrow\rangle$, but unconditional to the other nuclear spins. If k nuclear spins are present in the multi-nuclear spin register, 2^{k-1} ESR frequencies are needed to be driven in order to probe the state of a specific data qubit. After the state of the nuclear spin has been projected on the electron state, a final NMR π rotation conditional to $|e\rangle = |\uparrow\rangle$ is applied to prepare the nuclear spin to $\Downarrow$. Note that if $|n_1\rangle = |\Downarrow\rangle$ from the beginning, the sequence has no effect on the nuclear spin. To initialise all nuclear spins to $\Downarrow$, we perform such a EST sequence for each spin sequentially—with up to 3 repetitions if high fidelity is required. To reach an initialisation fidelity above 99%, we perform a QND nuclear state readout after the EST sequence and discard undesired states via post-selection.

VI. NUCLEAR STATE PREPARATION AND MEASUREMENT (SPAM) ANALYSIS

During QND nuclear-spin readout, there are cases in which the net spin signal $|P_{\Uparrow} - P_{\Downarrow}|$ is too small to unambiguously determine the spin state (see grey shaded region in Fig. S4b). To improve readout fidelity, we apply post-selection by retaining only those counts where $|P_{\Uparrow} - P_{\Downarrow}|$ exceeds a certainty threshold (typically ± 0.2).

Table S1 reports the rejection rate associated with this post-selection process for each nuclear spin. Within the 4P register, qubits n_1 and n_2 exhibit slightly higher rejection rates compared to n_3 and n_4, which we attribute to the spectral proximity of their conditional ESR transitions arising from similar hyperfine couplings. Overall, the post-selection rejection rates remain low, typically below 2.5%, indicating high classification confidence across the device.

#Qubit	Readout loss (%)
n_1	1.91
n_2	2.43
n_3	0.15
n_4	0.43
n_5	1.83
n_6	1.22
n_7	1.58
n_8	1.41
n_9	1.27

TABLE S1. **QND nuclear spin readout post-selection rejection rate.** Counts are accepted when the net spin signal satisfies $|P_{\Uparrow} - P_{\Downarrow}| \geq 0.2$. Rejection rates for individual qubits are extracted from the QND readout data shown in Fig. S4.

For the majority of experiments presented in the manuscript, we target the fully polarised nuclear state $\Downarrow$ for all spins. To achieve this, we perform EST sequentially for each nuclear spin. In addition, we decrease ESR Rabi frequencies to be 142 kHz and 100 kHz for the π rotations to reduce frequency crosstalk.

Table S2 shows the rejection rates associated with register-state classification and initialisation. The fraction of discarded counts from spin-state classification is comparable to that observed for single-qubit QND readout. By contrast, the initialisation procedure exhibits higher loss, which we attribute to frequency crosstalk arising during coherent ESR π-pulses. Given the high fidelity of nuclear-spin readout, we opt to post-select the initial state in all experiments presented in this work.

Register state	Readout loss (%)	initialisation loss (%)
$\Downarrow^4$	0.8(1)	7.4(1)
$\Downarrow^5$	0.6(2)	15(2)

TABLE S2. **Rejection rates for register-state classification and initialisation.** As in single-qubit QND readout, counts are accepted when the net spin signal satisfies $|P_{\Uparrow} - P_{\Downarrow}| \geq 0.2$. The states $\Downarrow^4$ and $\Downarrow^5$ correspond to all nuclear spins in the $\Downarrow$ state within the 4P and 5P registers, respectively. All values are extracted from the coherent Rabi data shown in Fig. 1 of the main text.

To highlight the robustness of the randomized benchmarking (RB) protocol against SPAM errors, Table S3 reports the primitive gate fidelities extracted from single-qubit and two-qubit RB experiments, both before and after applying initialisation post-selection and readout filtering. In all cases, the fidelities remain statistically equivalent within error bars, confirming that SPAM contributions are effectively decoupled from the RB estimates.

1Q-RB	Prior post-sel. (%)	After post-sel. (%)
n_1	99.979(3)	99.978(2)
n_2	99.982(3)	99.981(2)
n_3	99.971(2)	99.971(2)
n_4	99.603(2)	99.633(2)
n_5	99.988(4)	99.987(3)
n_6	99.979(2)	99.982(3)
n_7	99.969(2)	99.969(2)
n_8	99.947(2)	99.942(2)
n_9	99.938(2)	99.936(2)
**2Q-RB**		
Ref.	99.710(6)	99.731(5)
CZ	99.93(7)	99.90(4)

TABLE S3. **Nuclear randomized benchmarking fidelities.** Primitive gate fidelities extracted from single-qubit (1Q-RB) and two-qubit (2Q-RB) randomized benchmarking, reported before and after applying initialisation post-selection and the readout certainty threshold. Two-qubit benchmarking is performed on the qubit pair (n_6, n_9), with "Ref." referring to the reference sequence and "CZ" to the interleaved gate. The values shown in Fig. 1f (1Q-RB) and Fig. 2b (2Q-RB) of the main text correspond to the post-selected data.

VII. FREQUENCY STABILITY OF NUCLEAR MAGNETIC RESONANCES (NMR)

We also assess fluctuations of the NMR frequencies by repeatedly measuring NMR spectra. We first initialise the system in the $(\downarrow\Downarrow^4, \downarrow\Downarrow^5)$ configuration. Subsequently, we apply a specific NMR drive before finally reading the nuclear spin via QND readout. All nuclear spins in the 4P register shows fluctuations below 50 Hz (see Fig. S5a), except for n_4 which exhibits a non-negligible drift (up to 1.3 kHz) over 10 hours. We find that the observed NMR stability correlates with the hyperfine Stark coefficient, η, of the nuclear spins (see Fig. S6d). Owing to large η value of n_4 which is ≈ -25 MHz/(MV/m), this nuclear spin is more susceptible to charge noise. We can mitigate the impact of such a strong frequency drift by more frequent recalibrations of n_4. On the contrary, the 5P register shows no slow drifts (see Fig. S5b). However, a striking feature of this data is the correlated jump of the nuclear spins n_5, n_7 and n_8 (red shaded regions). We suspect that these correlated jumps stem from charge switches in the qubit environment slightly modifying the hyperfine interaction strengths and thereby the nuclear qubit frequencies. Note that, since these frequency jumps are less than 500 Hz in magnitude, any changes in hyperfine values are not sufficient to explain the 45 kHz jumps observed in the ESR frequency tracking data shown in Fig. S2b. We remark that for n_1, n_2, n_3 of the 4P register and n_6 of the 5P register, we find remarkable NMR stability with fluctuations below 50 Hz.

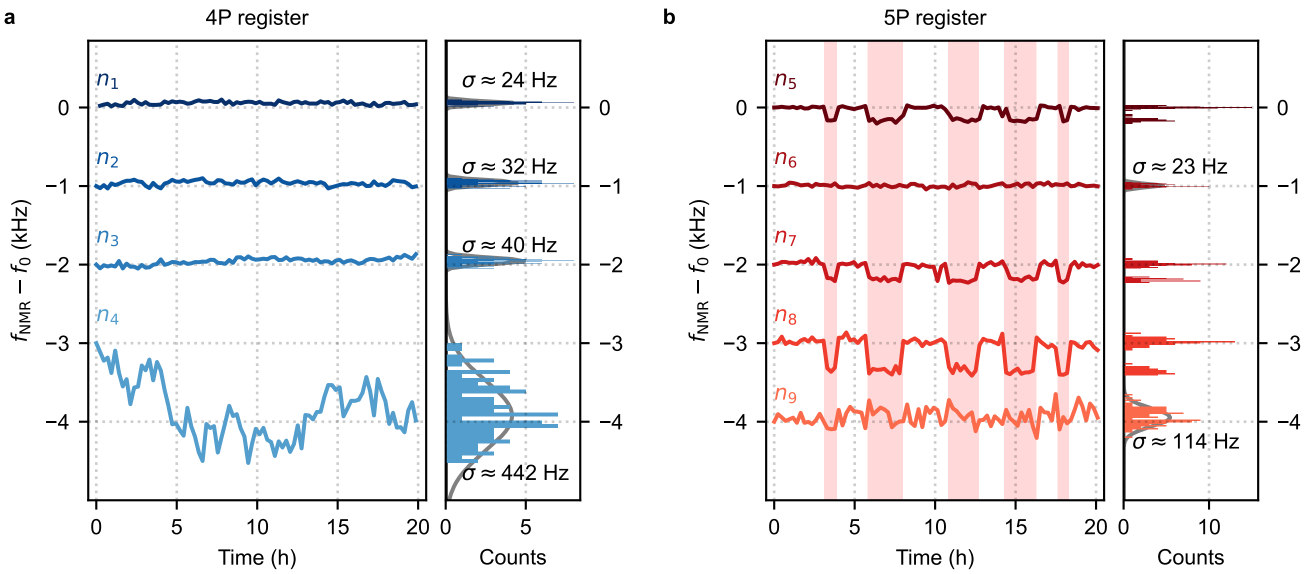

FIG. S5. **Stability of the NMR frequencies a (b),** Evolution of resonant NMR frequencies over time for nuclear spins in the 4P (5P) register. The first data point f_0 is subtracted to highlight relative changes and the traces are vertically shifted for clarity. Red shaded regions show correlated jumps between n_5, n_7 and n_8.

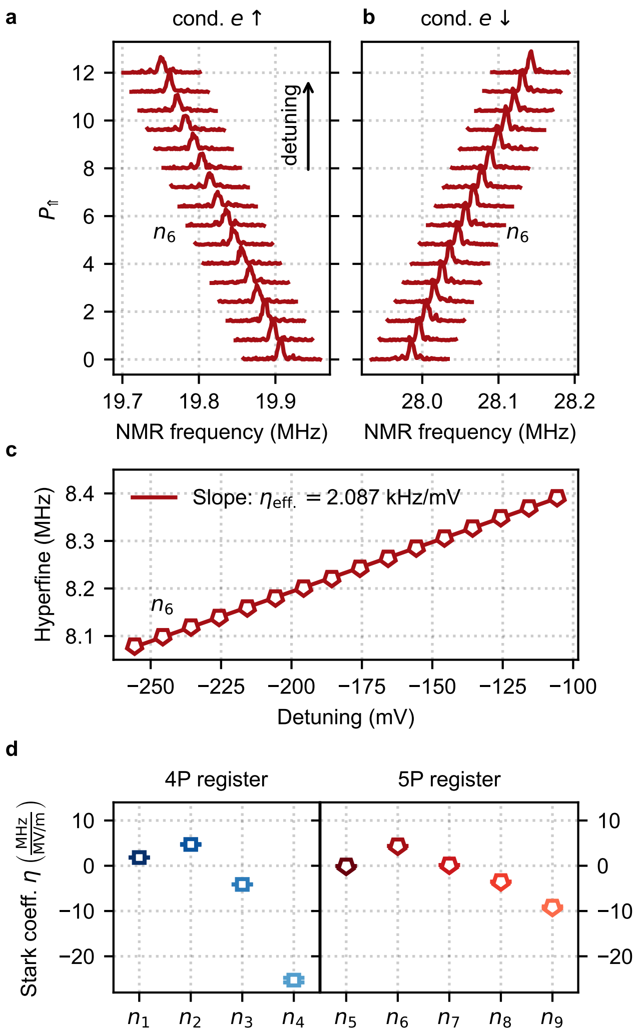

FIG. S6. **Hyperfine Stark shift—method and values for all nuclear spins. a (b),** Coherent NMR spectrum of n_6 conditional on the electron spin up-state ↑ (down-state ↓) as a function of detuning, ε. Traces are vertically shifted by 0.8 for clarity. **c,** The value of the hyperfine coupling in MHz extracted from NMR peak locations in **a** and **b**. The slope of the linear fit (solid line) represents the effective Stark coefficient, $\eta_{\mathrm{eff.}}$. **d,** Stark coefficient, $\eta = \eta_{\mathrm{eff}} \cdot d_{\mathrm{gate}}/\alpha$, obtained by normalizing η_{eff} with the electric field employing the distance between the gates G_1 and G_2 $d_{\mathrm{gate}} = 146.6$ nm and the V-to-eV conversion factor $\alpha = 0.07$.

VIII. OPTIMISATION OF GATE OPERATIONS

A. Compensation of frequency shifts induced by microwave drive

Microwave-induced frequency shifts during spin qubit operation in semiconductor devices have been repeatedly reported in the literature [10–12]. We investigate this effect in our multi-nuclear spin register by running Ramsey-like experiments with the nuclear- and electron-spin qubits in which we replace the idling between the $\pi/2$-pulses with off-resonant NMR (ESR) driving at 50 MHz (38.86 GHz). The period of the resulting Ramsey oscillations thus reflects the frequency shift that is introduced by the intermediate off-resonant drive. Figure S7 shows the corresponding frequency shift as function of the amplitude of the off-resonant driving power. The four panels show different cases where an ESR or NMR Ramsey pulse is executed with either ESR or NMR off-resonant driving. For example the panel "ESR on NMR" shows the results of an NMR-Ramsey experiment sandwiched with an off-resonant ESR drive of varying amplitude in mV.

We first focus on the influence of the NMR drive amplitude on the NMR resonance frequency—see Fig. S7a. Here we observe quadratic changes in the NMR frequency as we increase the power of the off-resonant NMR signal. In contrast to the work by Undseth *et al.* [11], we observe no sign of saturation over the range investigated. A striking feature of the "NMR on NMR" data is the different behavior for each of the registers: The nuclear spins of the 5P register show significantly smaller shifts. We suspect that the difference stems from the specific geometry of the phosphorus atoms within the multi-nuclear spin registers. Accordingly, we anticipate that advances in deterministic phosphorus atom placement will enable us to reduce the susceptibility to NMR driving. For all the other cases "ESR on NMR", "NMR on ESR" and "ESR on ESR"—see Fig. S7b-d—, we observe smaller impact on qubit operation. For electron spins e_1 and e_2, we observe a non-monotonous shifts in ESR frequency what poses a challenge for consistent operation. In general we find that the extent of the ESR and NMR shifts increase with ESR or NMR driving power.

Overall, the data shows that both ESR and NMR qubit frequencies change during any idling time if the total microwave input power is not kept constant during circuit operations. To compensate for such coherent errors, we equalise the effective input power applied at any time by calibrating the electron (nuclear) qubit frequencies with parallel NMR (ESR) drive at an off-resonant frequency, $f_{\text{off}}^{n/e}$, as shown in the circuit below:

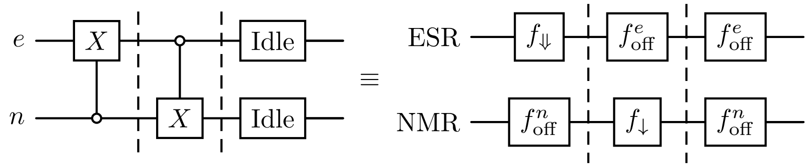

We thus establish a fixed budget for the antenna drive that covers the effective load for both ESR and NMR. In this work, we set $f_{\text{off}}^{n} = 50$ MHz and $f_{\text{off}}^{e} = 38.86$ GHz. This approach ensures consistent input power during antenna operation with any partial or complete idling periods.

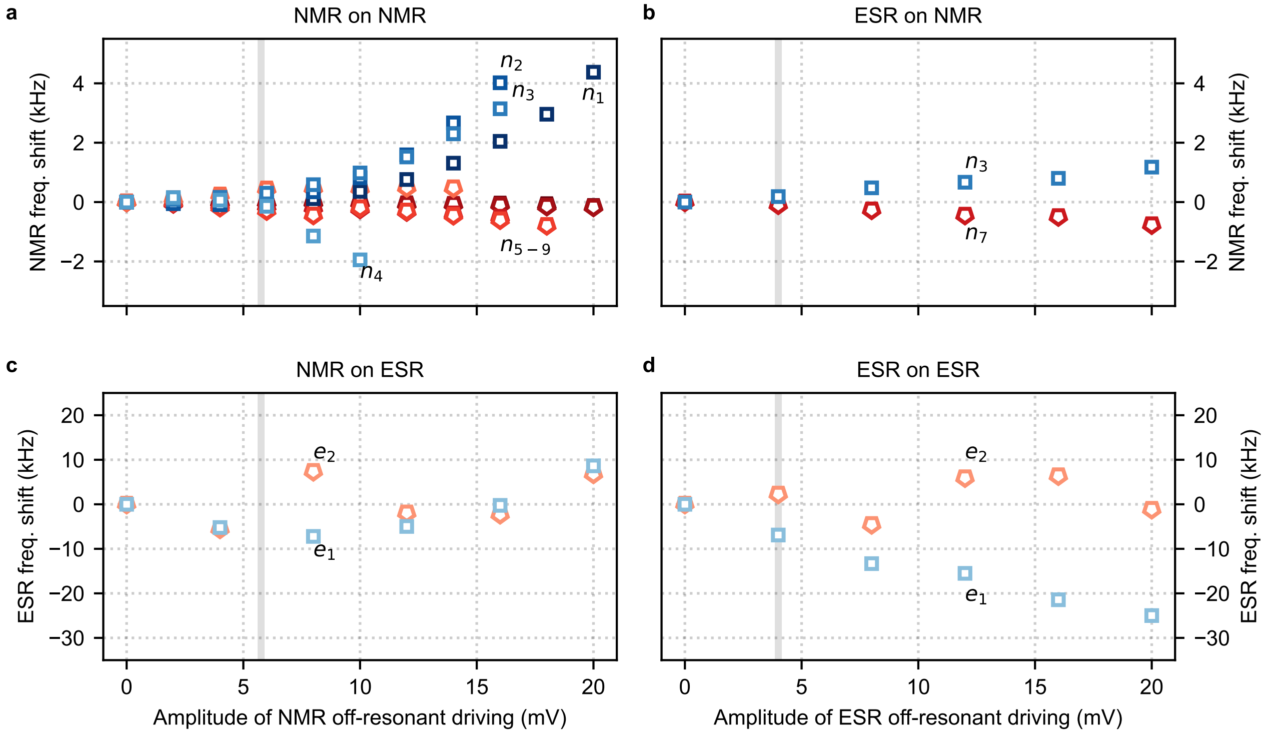

FIG. S7. **Shifts in electron and nuclear spin-qubit frequencies with off-resonant microwave drive. a,** Frequency-shift of a nuclear-magnetic-resonance (NMR) peaks by off-resonant NMR drive ("NMR on NMR") with increasing amplitude. The frequency shifts are recorded via nuclear Ramsey experiments with off-resonant NMR drive at $f_w = 50$ MHz during the idling time. An initial microwave burst of 500 μs at f_w is applied before the Ramsey pulse and a waiting time of 2 ms before the readout. The off-resonant drive amplitude is the applied peak-to-peak voltage at room temperature without taking into account attenuation. **b,** Frequency-shift of NMR peaks by an off-resonant electron-spin-resonance (ESR), namely "ESR on NMR", with increasing amplitude. Similar experiment to **a**, but using instead an off-resonant ESR drive at $f_w = 38.86$ GHz during the idling time. Here, we have only characterised one nuclear spin from each register, n_4 (4P) and n_7 (5P), as the impact on our measurements is comparably small in this case. **c,** Frequency-shift of ESR peaks by an off-resonant NMR drive ("NMR on ESR") with increasing amplitude. Similar experiment than in **a**, but performing an electron Ramsey sequence with NMR off-resonance driving during idling. **d,** Frequency-shift of ESR peaks by an off-resonant ESR drive ("ESR on ESR") with increasing amplitude. For the present experiment, we set the off-resonant NMR and ESR amplitudes to 5.75 mV and 4 mV (shaded grey).

B. Optimisation of NMR Rabi frequencies

The amplitude of the NMR pulses determines the corresponding Rabi frequency f_{Rabi} and is thus important for efficient operation. Faster rotations make it possible to carry out more operations before error sources have an impact. On the other hand, stronger NMR drive causes shifts of NMR and ESR frequencies—see Fig. S7. Accordingly, the optimal choice of the NMR drive power needs to balance these aspects. Here, we use the fidelity of 1Q-RB experiments as metric to find the optimal value of f_{Rabi}.

For the 4P register, the resulting 1Q-RB data—see Fig. S8a—shows the effect of NMR power on the gate fidelity for all nuclear spins. Above a drive amplitude of 4 mV, where maximal fidelity >99.95% is observed, the Larmor frequency starts to shift with a concomitant drop in performance. This observation is not true for n_4 which shows an optimal performance at 6 mV. For the range of drive amplitudes investigated, we observe a linear dependency on the Rabi frequency and no change on T_2^* (data not shown). For the 5P register, no drop in 1Q-RB fidelity is observed as the NMR-drive amplitude is increased—see Fig. S8b. The data is consistent with the shifts in nuclear spin-qubit frequencies induced by off-resonant NMR drive which saturates at a smaller magnitude—compare pentagon markers in Fig. S7a.

The NMR amplitudes for maximal 1Q-RB fidelity only remain a good choice if the absorbed power, which for NMR is proportional to the ratio of the Rabi and Larmor frequency $f_{\mathrm{Rabi}}/f_{\mathrm{NMR}}$, is constant. Otherwise, if multiple NMR pulses are employed, the power budget varies during execution owing to the different shifts in the Larmor frequency. We plot the Rabi coupling versus the NMR-Larmor frequency in Fig. S8c. We find a deviation from a linear trend for drive frequencies beyond 30 MHz. For NMR pulses driven beyond this threshold, we therefore need to increase the drive amplitude to keep the ratio of $f_{\mathrm{Rabi}}/f_{\mathrm{NMR}}$ constant. The corresponding increase in drive amplitude is extracted via a polynomial fit of degree 3 (dashed line) to the data. A list of typical NMR drive parameters after such adjustments are listed in Table S4. The Table is sorted by NMR-Larmor frequency. The off-resonant NMR compensation pulse—for idling —is set to $f_{\mathrm{NMR}} = 50$ MHz and $A_{\mathrm{NMR}} = 5.421$ mV to achieve similar power absorption to the parameters listed. We note that this procedure cannot be generalised for ESR owing to a different relation of the absorbed power to the Rabi and Larmor frequency.

**#Qubit**	f_{NMR} **(MHz)**	f_{Rabi} **(kHz)**	A_{NMR} **(mV)**
n_1	24.450	3.582	4.726
n_2	24.534	3.594	4.725
n_3	24.985	3.661	4.750
n_4	65.412	9.584	5.895
n_5	24.221	3.538	4.789
n_6	28.110	4.118	4.783
n_7	34.324	5.029	4.952
n_8	43.902	6.432	5.327
n_9	123.026	18.025	6.393

TABLE S4. **Optimised NMR-drive parameters.** To ensure the same power absorption, we keep the ratio $f_{\mathrm{Rabi}}/f_{\mathrm{NMR}} = 1.46 \times 10^{-4}$ constant.

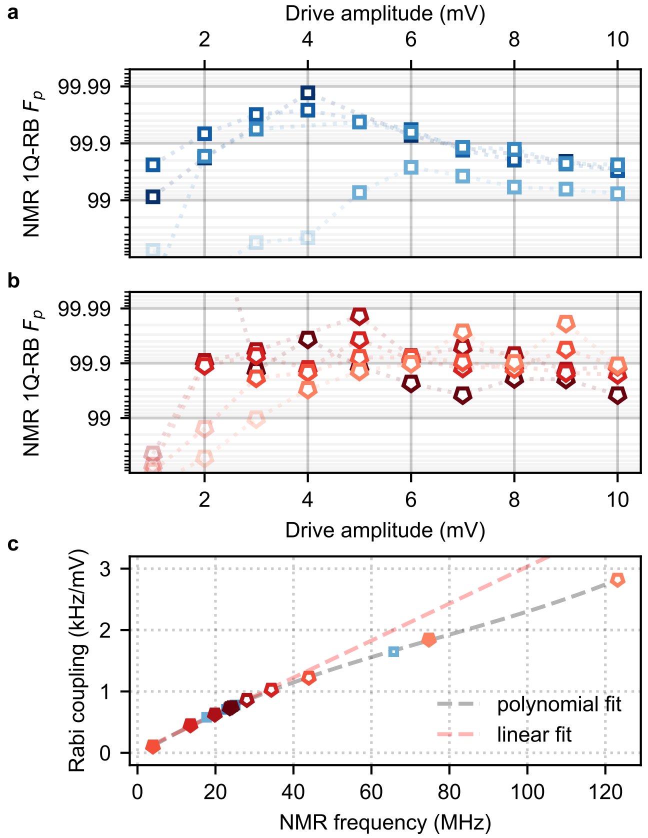

FIG. S8. **Optimisation of nuclear-spin drives via single-qubit randomised benchmarking (1Q-RB). a (b),** Primitive 1Q-RB fidelity as a function of the room-temperature drive amplitude for nuclear spins in the 4P (5P) register. Data points above 99% are highlighted. **c,** Rabi coupling, $f_{\mathrm{Rabi}}/A_{\mathrm{drive}}$, for coherent NMR frequencies conditional to electron down-state $\downarrow$ (open symbols) and up-state $\uparrow$ (closed symbols). The data points are extracted using Rabi frequencies f_{Rabi} from **a** and **b**. Grey dashed line is an empirical fit using a polynomial function of degree 3. To highlight the linearity between f_{Rabi} and f_{NMR}, data points below 40 MHz are fitted with a linear function (red dashed line).

C. Optimisation of the electron-exchange-based CROT operation

To link multi-nuclear spin registers we operate an exchange-based CROT gate [13, 14] at low exchange coupling $J \sim 1.5 - 1.7$ MHz, *i.e.,* much smaller than the Larmor-frequency splitting of the CROT resonances of e_1 and e_2 of $\Delta E_z \approx 109.9$ MHz. In this regime, where $J \ll \Delta E_z$, the CROT operation is less susceptible to charge noise and not conditional on the nuclear spin states of the other multi-nuclear spin register.

To further optimise the fidelity of the CROT operation, we can maximise its driving speed given by the Rabi frequency f_{Rabi}. Such an increase in Rabi speed, however, comes with power broadening since for $f_{\mathrm{Rabi}} \gtrsim J/4$ off-resonant drive of the other exchange resonance zCROT becomes significant and causes population transfer to the neighboring resonant peak. This effect can be particularly strong if no pulse shaping is applied (as in this work). We can, however, mitigate the off-resonance drive if we choose the Rabi frequency such that the neighboring zCROT peak lies within a node of the power spectrum. The spin-flip probability (P_s) of an antenna drive without any pulse modulation (*i.e.* boxcar window), follows the the function:

$$P_s = \frac{\Omega^2}{\Omega^2 + \Delta^2} \cdot \sin^2\left(\frac{t\sqrt{\Omega^2 + \Delta^2}}{2}\right) \tag{5}$$

where $\Omega = 2\pi f_{\mathrm{Rabi}}$ is the angular Rabi frequency, $\Delta = 2\pi\delta f$ is the angular frequency offset with respect to the Larmor frequency and t is the duration of the pulse. This function has local minima for a specific set of Rabi frequencies:

$$f_{\mathrm{Rabi}}(n) = \sqrt{\frac{n^2}{t^2} - \delta f^2} \tag{6}$$

where n is an integer number. Optimizing f_{Rabi} for $\pi/2$ operations, we can minimise any off-resonant driving as shown in Fig. S9. For a $\pi/2$ rotation—with the duration $t = 1/(4 \cdot f_{Rabi})$—, we obtain:

$$f_{\mathrm{Rabi}}(n) = \delta f/\sqrt{16n^2 - 1}. \tag{7}$$

To fulfill this condition and obtain a maximum Rabi frequency, we set $n = 1$ for the discussed zCROT off-resonant drive of a CROT operation where $\delta f = J \approx 1.55$ MHz (1.69 MHz). Accordingly, we can minimise the off-resonant drive with a maximal speed at $f_{\mathrm{Rabi}}(n = 1) \approx 400$ kHz (436 kHz).

For the electron 2Q-RB data shown in Fig. 2 of the main paper, we also compensate for controlled-phase errors following the protocol defined by Ref. [15]. The data obtained from measurements of phase-shift characterisation for the CROT operation are shown in Fig. S10a. Applying the correction protocol, we observe a significant improvement in the standard and interleaved 2Q-RB as shown in Fig. S10b with all fidelity benchmarks above 99%. We remark however that by employing the electron spins as ancillary qubits, *i.e.,* as an entanglement mechanism between the nuclear spins, such phase errors have negligible impact, and thus no corrections are needed.

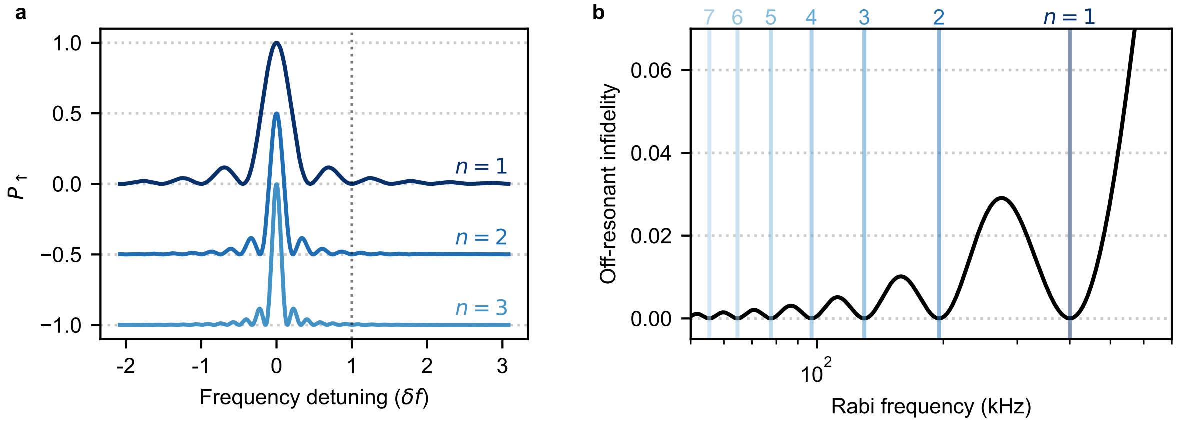

FIG. S9. **Optimisation of the Rabi frequency for high-fidelity CROT operation. a,** Analytical spin-flip probability for π rotations for various $f_{\mathrm{Rabi}}(n) = \delta f/\sqrt{16n^2 - 1}$ where $n \in [1, 2, 3]$. The traces are vertically shifted for better visualisation. Here, for exchange operations, the CROT resonance frequency is located at $\delta f = J$—see dotted line. **b,** Infidelity, $1 - P_\uparrow$, at a frequency detuning of δf as function of Rabi frequency. The minima correspond to optimal Rabi frequencies $f_{\mathrm{Rabi}}(n)$ for π rotations (vertical lines) where off-resonant drive is minimised at δf.

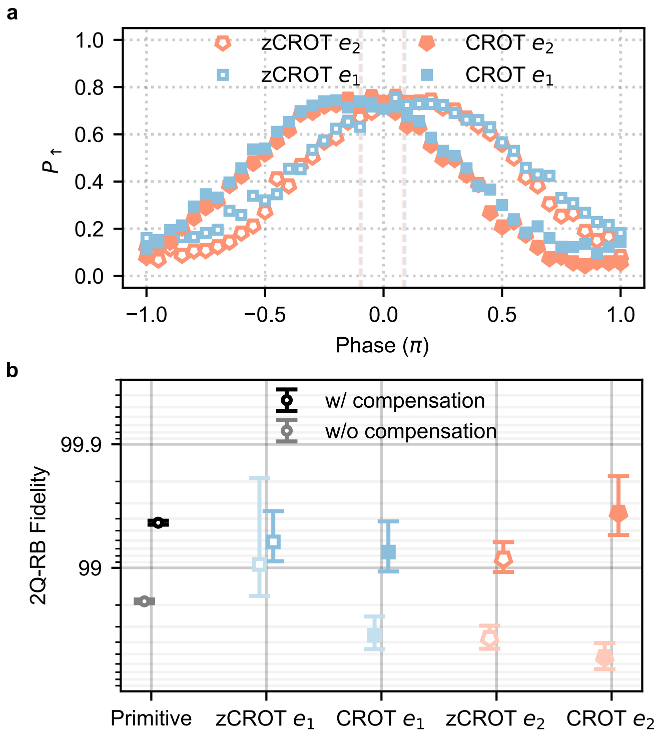

FIG. S10. **Optimisation of electron two-qubit randomised benchmarking (2Q-RB). a,** Phase shifts of coherent CROT operations following calibration sequences from Ref. [15]: We execute a Ramsey-type experiment with $\pi/2$-zCROT (CROT) pulses sandwiching the complementary π-CROT (zCROT) pulse under test with the other electron being correspondingly initialised to $\downarrow$ and $\uparrow$. The measurement is executed by sweeping the phase of the second $\pi/2$ pulse. **b,** e-e 2Q primitive (black) and interleaved RB fidelities (colors) with (solid) and without (semi-transparent) phase error compensation.

D. Optimal CROT gate speed for CZ operation

The smallest separation between two ESR transitions within a register sets the upper bound for the gate speed via the Eq. 7, which is typically defined by the exchange interaction J. However, in the presence of hyperfine couplings smaller than J, f_{Rabi} has to be reduced to mitigate off-resonant driving. For ESR rotations that implements nuclear CZ gates, we choose the optimal f_{Rabi} corresponding to the smallest hyperfine coupling involved in the nuclear 2Q gate. Table S5 lists the ESR drive speeds of e_1 and e_2 for nuclear CZ gates involving each nuclear spin.

**Qubit**	A **(MHz)**	f_{Rabi} **(kHz)**
n_1	1.0	258
n_2	1.1	284
n_3	2.2	436
n_4	83.6	436
n_5	0.6	154
n_6	8.2	436
n_7	20.8	436
n_8	40.0	436
n_9	197.8	436

TABLE S5. **Optimised ESR Rabi frequencies f_{Rabi} for nuclear CZ gate operations**. Optimal f_{Rabi} is calculated based on Eq. 7 for each data qubit where δf is the minimum between the hyperfine coupling A and the exchange coupling $J \approx 1.69$ MHz.

IX. BELL-STATE TOMOGRAPHY WITH REDUCED PROJECTIONS

To assess the accuracy of fidelity estimates from a reduced tomography scheme [16], we first extract the Bell-state fidelity using the complete set of projections. We then re-analyze the same dataset, restricting it to only the reduced set of three Pauli projections (XX, YY, and ZZ), and calculate the corresponding fidelity. Figure S11 shows the extracted fidelities for the two exemplary local and non-local Bell states shown in Fig. 3c and 3g in the main manuscript. We find that the resulting values agree within their respective uncertainty margins, indicating that the reduced protocol yields comparable accuracy in this context.

a Local Bell State (n_6, n_9)

	**Full QST Fid. (%)**	**Reduced QST Fid. (%)**
Φ^+	99.3(2)	99.3(2)
Ψ^+	99.3(1)	99.2(1)
Φ^-	99.3(2)	99.3(1)
Ψ^-	98.9(2)	98.9(2)
Avg.	99.2(3)	99.2(3)

b Non-Local Bell State (n_4, n_9)

	**Full QST Fid. (%)**	**Reduced QST Fid. (%)**
Φ^+	97.7(2)	97.8(2)
Ψ^+	95.9(3)	96.1(2)
Φ^-	97.7(2)	97.7(2)
Ψ^-	97.5(2)	97.5(3)
Avg.	97.2(9)	97.3(9)

FIG. S11. **Comparison of Bell state fidelities.** Fidelities of **a,** the local and **b,** the non-local Bell states ($\Phi^{\pm}$ and $\Psi^{\pm}$) estimated from Quantum State Tomography (QST) using the full or the reduced set of projections.

[1] D. Keith *et al.*, Ramped measurement technique for robust high-fidelity spin qubit readout, Science Advances **8**, 0455 (2022).
[2] S. J. Hile *et al.*, Addressable electron spin resonance using donors and donor molecules in silicon, Science Advances **4**, eaaq1459 (2018).
[3] I. Thorvaldson *et al.*, Grover's algorithm in a four-qubit silicon processor above the fault-tolerant threshold, Nature Nanotechnology **20**, 472–477 (2025).
[4] G. Waldherr *et al.*, Quantum error correction in a solid-state hybrid spin register, Nature **506**, 204–207 (2014).
[5] M. T. Mądzik *et al.*, Precision tomography of a three-qubit donor quantum processor in silicon, Nature **601**, 348–353 (2022).
[6] M. T. Mądzik *et al.*, Conditional quantum operation of two exchange-coupled single-donor spin qubits in a MOS-compatible silicon device, Nature Communications **12**, 1 (2021).
[7] D. Holmes *et al.*, Improved Placement Precision of Donor Spin Qubits in Silicon using Molecule Ion Implantation, Advanced Quantum Technologies **7**, 2300316 (2024).
[8] J. J. Pla *et al.*, High-fidelity readout and control of a nuclear spin qubit in silicon, Nature **496**, 334–338 (2013).
[9] S. Monir *et al.*, Impact of measurement backaction on nuclear spin qubits in silicon, Physical Review B **109**, 035157 (2024).
[10] S. G. J. Philips *et al.*, Universal control of a six-qubit quantum processor in silicon, Nature **609**, 919–924 (2022).
[11] B. Undseth *et al.*, Hotter is Easier: Unexpected Temperature Dependence of Spin Qubit Frequencies, Physical Review X **13**, 041015 (2023).
[12] T. Tanttu *et al.*, Assessment of the errors of high-fidelity two-qubit gates in silicon quantum dots, Nature Physics **20**, 1804–1809 (2024).
[13] R. Kalra, A. Laucht, C. D. Hill, and A. Morello, Robust Two-Qubit Gates for Donors in Silicon Controlled by Hyperfine Interactions, Physical Review X **4**, 021044 (2014).
[14] L. Kranz *et al.*, High-Fidelity CNOT Gate for Donor Electron Spin Qubits in Silicon, Physical Review Applied **19**, 024068 (2023).
[15] Y.-H. Wu *et al.*, Hamiltonian phase error in resonantly driven CNOT gate above the fault-tolerant threshold, npj Quantum Information **10**, 8 (2024).
[16] O. Gühne, C.-Y. Lu, W.-B. Gao, and J.-W. Pan, Toolbox for entanglement detection and fidelity estimation, Phys. Rev. A **76**, 030305 (2007).
